# Systematic analysis and comparison of the burden of cardiovascular diseases in China from 1990 to 2021 and its 15-year prediction with global levels

**DOI:** 10.3389/fcvm.2025.1660259

**Published:** 2025-12-04

**Authors:** Tian-ming Gan, Honghong Ke, Yingjie Yang, Guan-lian Mo, Shi-rong Wang, Jin-yi Li

**Affiliations:** 1 Department of Cardiovascular Medicine, The First Affiliated Hospital of Guilin Medical University, Guilin, China; 2 Department of Cardiovascular Medicine, The First Affiliated Hospital of Guangxi Medical University, Nanning, China

**Keywords:** cardiovascular diseases, disease burden, China, global, gender disparities

## Abstract

**Background and aims:**

Systematic assessment of cardiovascular disease (CVD) risk factors is crucial for prevention. We used GBD 2021 data to examine long-term trends and epidemiological features of CVDs in China and globally.

**Methods and results:**

CVD prevalence, incidence, mortality, and DALYs were analyzed using joinpoint regression, age-period-cohort models, decomposition, ARIMA, and BAPC. From 1990 to 2021, age-standardized mortality and DALY rates declined, but prevalence and case numbers increased, with males bearing a higher burden.

**Conclusion:**

Rapid population aging sustains high CVD burden, highlighting the urgent need for targeted prevention and management strategies.

## Introduction

Cardiovascular diseases (CVDs), which affect the heart and blood vessels, are the leading cause of death globally and impose tremendous social and economic costs. Risk factors include hypertension, high cholesterol, smoking, obesity, diabetes, and physical inactivity (1, 2). CVDs are not only a serious problem in high-income countries; over 80% of CVD cases and related deaths occur in low- and middle-income countries (3). In China, CVDs account for more than 40% of deaths (4–6). Predominantly affecting middle-aged and older adults, CVDs have become a significant public health issue worldwide, exacerbated by the increasing aging population (7, 8). Therefore, it is crucial to halt the rise in prevalence and incidence and minimize their overall impact on public health. China is one of the countries with a heavy burden of CVDs, facing significant challenges in addressing this issue. In 2011, the United Nations identified non-communicable diseases, including CVDs, as a major global health problem, with an action plan aimed at reducing premature deaths by 25% by 2025, focusing on prevention, early detection, and treatment, as well as addressing risk factors such as unhealthy diets, lack of physical activity, smoking, and alcohol consumption (9, 10). Therefore, understanding the CVD burden in Chinese and global populations across different periods can help formulate more targeted health policies to reduce disease risks.

However, while previous studies have systematically analyzed the burden of CVDs from 1990 to 2019, existing research has limited scope, focusing only on single CVD subcategories. More analysis of temporal changes is needed to provide additional foundational knowledge for decision-making. Systematic assessment of data collected from other available sources can provide valuable decision support for policymakers, identify effective disease control strategies to minimize the burden, and help fill knowledge gaps.

The Global Burden of Diseases (GBD) study is a multinational collaborative research effort aimed at estimating the burden of various diseases and their contributing factors at the global, regional, and national levels. Studies analyzing the burden of CVDs have been conducted worldwide. However, specific analyses assessing the CVD burden at the national level require more comprehensive coverage. Using data from 1990 to 2021, this study analyzed the overall burden of CVDs (prevalence, incidence, mortality, and DALYs rates) in China and globally. This research will describe the differing trends in the CVD burden in China and globally. The aim is to provide health authorities with an assessment of the existing CVD burden and its changing trends. This information will enable health authorities to prioritize future public health initiatives and healthcare services. Our findings provide evidence-based data for CVD prevention.

## Methods

### Data source and preprocessing

The GBD database primarily utilizes DisMod-MR 2.1 (Disease Modeling Meta-Regression, version 2.1) as its estimation methodology. DisMod-MR 2.1 is a Bayesian disease modeling meta-regression tool that generates internally consistent estimates of incidence, prevalence, and mortality based on relevant data. This study used the latest GBD 2021 data, which were directly downloaded from the official website of the Global Health Data Exchange (GHDx) (https://ghdx.healthdata.org/). Minimal preprocessing included checking file locations and ensuring consistency of file labels.

The database provides detailed records of incidence, prevalence, mortality, and DALYs rates for over 300 diseases and injuries, classified by age and sex, across 204 countries and regions. Since the data are publicly available, specific ethical approval was not required.

Potential biases inherent in the GBD data, such as uneven data availability across countries, variations in disease coding, and modeling assumptions, may affect the estimates. These issues are partially mitigated by the DisMod-MR 2.1 framework, which generates internally consistent estimates and provides 95% UIs. In our descriptive analyses, we utilized the provided 95% UIs to reflect uncertainty.

### Statistical analysis

Statistical analysis and visualization of the study data were conducted using R software (version 4.3.1) and Joinpoint software (version 5.1.0.0). A *p*-value < 0.05 was considered statistically significant.

### Descriptive analysis

We conducted a descriptive analysis of the temporal trends in the burden of CVDs in China and globally.

### Joinpoint regression analysis

We used Joinpoint software to calculate the annual percent change (APC), average annual percent change (AAPC), and corresponding 95% confidence intervals (95% CIs) for the incidence, prevalence, mortality, and DALYs rates of CVDs in China and globally from 1990 to 2021. The most suitable models were selected for comparison, and the trends in disease burden were assessed. An AAPC estimate with a 95% CI greater than 0 indicates an upward trend; less than 0 indicates a downward trend; and equal to 0 indicates a stable trend.

### Age-period-cohort analysis

A web-based age-period-cohort analysis tool was run in *R* to analyze the impact of age, period, and cohort on the prevalence of cardiovascular diseases (CVDs).The Bayesian age-period-cohort (BAPC) model incorporated second-order random walk priors for age, period, and cohort effects, which impose smoothness and reduce overfitting. The probabilistic predictions obtained were well-calibrated, with credible intervals reflecting uncertainty, indicating that the model provided relatively precise predictions. Age was assessed by examining the influence of age-related factors; period represented changes in anthropogenic factor loads during specific time periods; and cohort was associated with differences in exposure conditions among populations born in different periods. To address the issue of multicollinearity between age, period, and cohort, Poisson-based intrinsic estimators were used to derive disease parameters in the model. To prevent information overlap between adjacent cohorts, the duration, time range, and time intervals for cohorts were consistent. Therefore, the intervals for age, period, and birth cohort were all set to 5 years. Net drift represented the overall time trend, with *p* < 0.05 considered statistically significant. The longitudinal age curve was used to assess changes in disease burden attributed to age effects. The period rate ratio (RR) and cohort RR revealed the period and birth cohort effects, respectively, with RR > 1 indicating an increased relative risk of disease and RR < 1 indicating a decreased risk, compared to the reference cohort.

### Decomposition analysis

Das Gupta's decomposition method was employed to investigate the impact of population growth, population aging, and epidemiological trends on the burden of CVDs. This method, a classic one commonly used in decomposition analysis, utilizes mathematical techniques to decompose overall changes into their constituent parts to determine the specific contribution of each. Using this method, we studied the impact of age structure, population growth, and epidemiological changes on the burden of CVDs in China and globally.

In the decomposition analysis, the change in each GBD metric (prevalence, incidence, deaths, and DALYs) from 1990 to 2021 was partitioned into three components: (1) Aging effect, capturing the impact of changes in population age structure; (2) Population effect, capturing the impact of changes in total population size; and (3) Epidemiological change, capturing changes in age-specific rates (prevalence, incidence, mortality, or DALY rates) independent of population size and age structure.

### ARIMA model for burden prediction

The Autoregressive Integrated Moving Average (ARIMA) model is a widely used time series analysis method that combines autoregressive (AR), integrated (I), and moving average (MA) components to effectively capture trends and cyclic patterns in time series data and predict future changes based on existing data. In the ARIMA (p, d, q) model, “p” represents the number of autoregressive terms, “d” represents the number of differencing terms, and “q” represents the number of moving average terms. We first stabilized the time series data using differencing and confirmed stationarity using the Kwiatkowski-Phillips-Schmidt-Shin (KPSS) test. Then, Q-Q plots were used to assess whether the residuals followed a normal distribution. Next, we compared the goodness of fit of different models using the Akaike Information Criterion (AIC) and Bayesian Information Criterion (BIC), selecting the model with the smallest criterion value. Finally, the Ljung-Box test was used to verify the robustness of the residuals. When the residuals of the ARIMA model exhibit randomness (white noise), the model is considered the best linear predictor for short-term time series forecasting.

### BAPC model for burden prediction

The BAPC model is developed based on the age-period-cohort model, assuming a correlation between incidence or mortality and age structure and population size. The BAPC model combines second-order random walks to smooth the priors for age, period, and cohort effects to predict future incidence or mortality. This method incorporates nested Laplace approximations, avoiding any mixing and convergence issues caused by sampling techniques associated with Markov Chain Monte Carlo, demonstrating improved coverage and accuracy compared to other methods. Based on age-specific population data from 1990 to 2021, predicted population data for 2022–2035, and the GBD world population age standard, the BAPC model was used to predict the ASIR (Age-Standardized Incidence Rate), ASPR (Age-Standardized Prevalence Rate), ASMR (Age-Standardized Mortality Rate), and ASDR (Age-Standardized Disability-Adjusted Life Years Rate) of CVDs in China and globally for the next 15 years.

### Model validation and sensitivity analysis

To assess the robustness and predictive reliability of our models, we performed sensitivity analyses and cross-validation for both the ARIMA and BAPC models. Specifically, we used historical data from 2015 to 2018 to predict the years 2019–2021, and compared the predicted values with the actual observed data. The predictive performance was evaluated using standard statistical indicators, including the coefficient of determination (*R*^2^), root mean square error (RMSE), and mean absolute error (MAE). Relative errors were calculated to further assess the accuracy of short-term forecasts. All analyses were conducted separately for males and females in China and globally, across four main indicators: prevalence, incidence, mortality, and DALYs.

## Results

### Descriptive analysis

**Figure 1:**
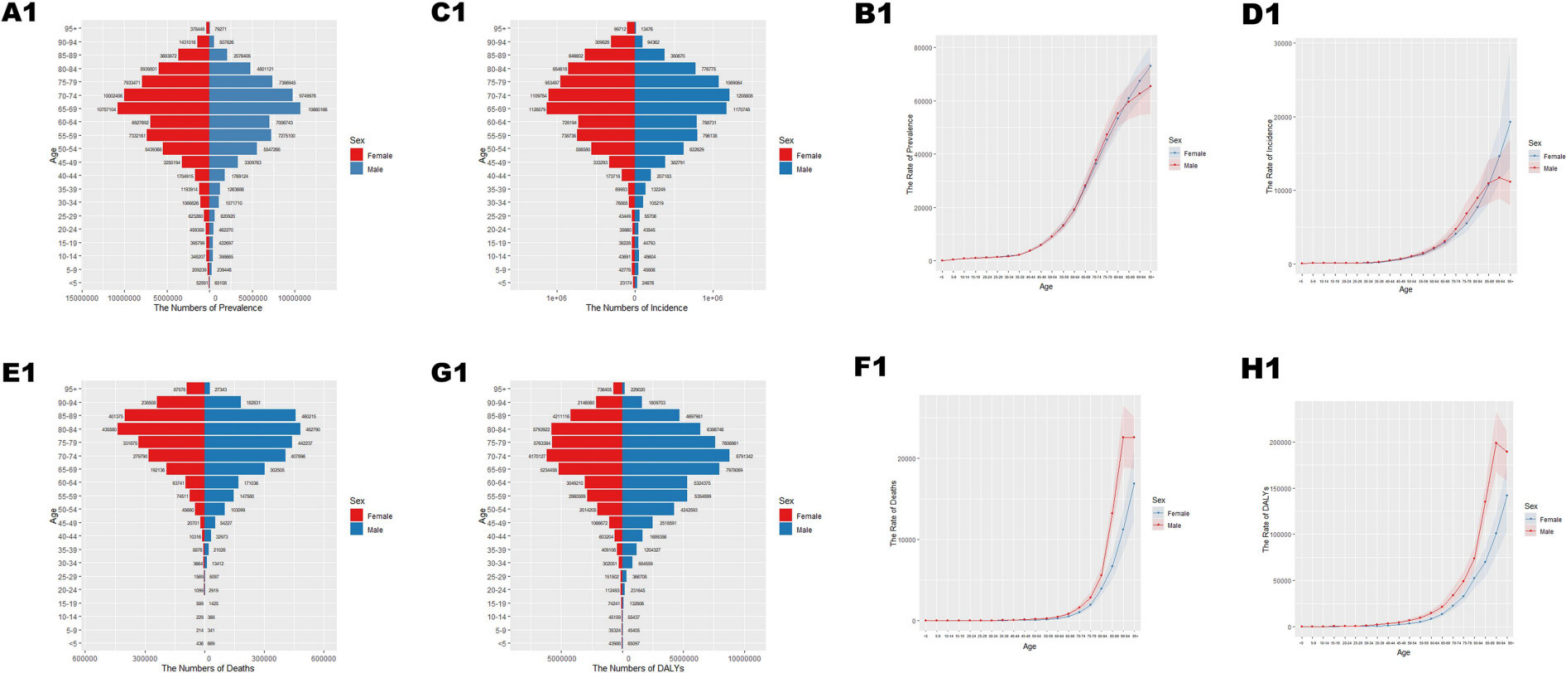
Age-specific numbers and age-standardized prevalence, incidence, and mortality of Cardiovascular Disease (CVD) in China in 2021. **(A1)** Number of prevalent cases by age. **(B1)** Age-standardized prevalence. **(C1)** Number of incident cases by specific age. **(D1)** Age-standardized incidence. **(E1)** Number of deaths by age. **(F1)** Age-standardized mortality rate. **(G1)** Disability-adjusted life years (DALYs) by age. **(H1)** Age-standardized DALYs.

**Figure 2:**
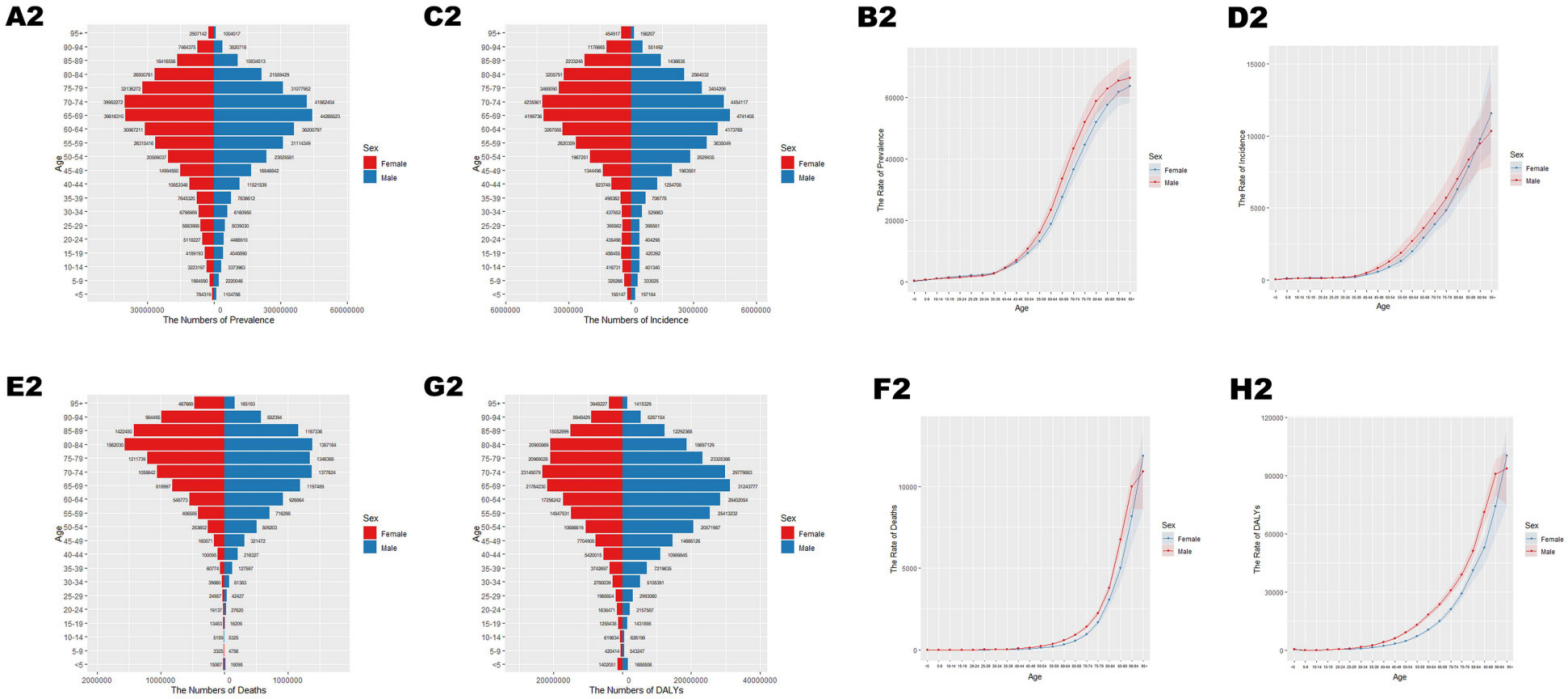
Age-specific numbers and age-standardized prevalence, incidence, and mortality of CVD globally in 2021. **(A2)** Number of prevalent cases by age. **(B2)** Age-standardized prevalence. **(C2)** Number of incident cases by specific age. **(D2)** Age-standardized incidence. **(E2)** Number of deaths by age. **(F2)** Age-standardized mortality rate. **(G2)** DALYs by age. **(H2)** Age-standardized DALYs.

**Figure 3:**
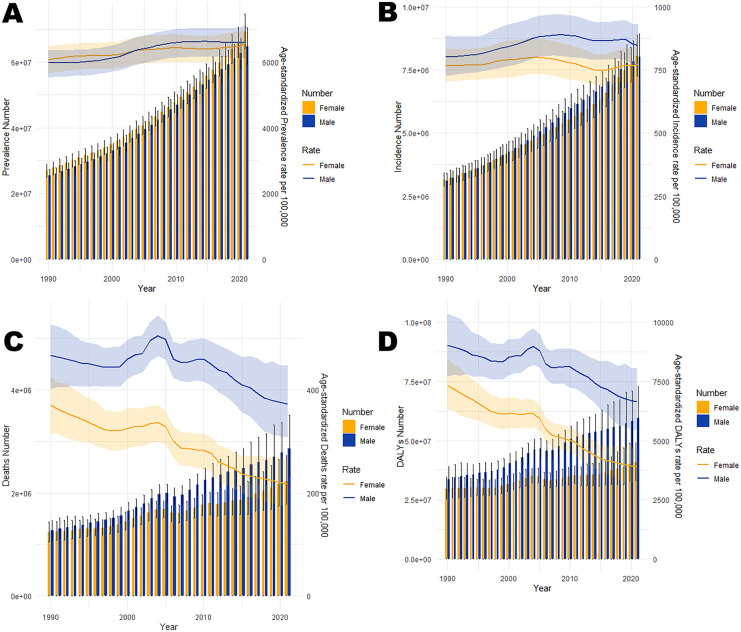
Trends in the all-age cases and age-standardized prevalence, incidence, mortality, and DALYs rates of CVD by gender in China from 1990 to 2021. **(A)** Number of prevalent cases and prevalence rate. **(B)** Number of incident cases and incidence rate. **(C)** Number of deaths and mortality rate. **(D)** Number of DALYs and DALYs rate.

**Figure 4:**
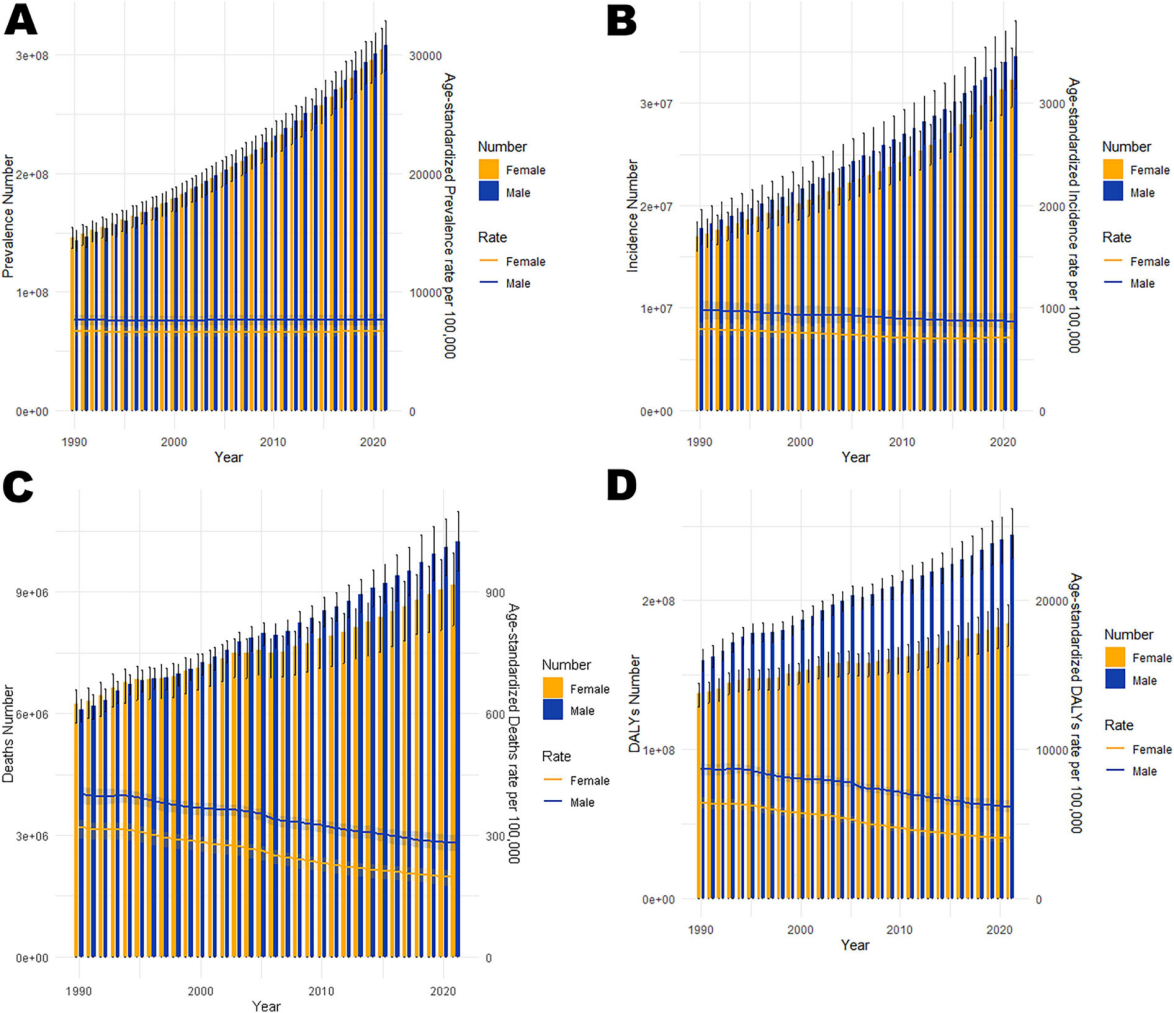
Trends in the all-age cases and age-standardized prevalence, incidence, mortality, and DALYs rates of CVD by gender globally from 1990 to 2021. **(A)** Number of prevalent cases and prevalence rate. **(B)** Number of incident cases and incidence rate. **(C)** Number of deaths and mortality rate. **(D)** Number of DALYs and DALYs rate.

In 2021, cardiovascular disease (CVD) indicators in China and globally showed significant age-related changes. In China (Table 1; Figure 1A), the number of patients peaked in those aged ≥65 with widened gender disparity, where females over 90 exceeded males. Age-standardized prevalence rate (ASPR) rose rapidly at ∼40 years, with a higher impact on females after 85. New cases (Table 1; Figure 1B) peaked at 70–74 years in males and 65–69 years in females, with female age-standardized incidence rate (ASIR) exceeding males after 85. Mortality cases (Table 1; Figure 1C) peaked at 80–84 years, with higher male age-standardized mortality rate (ASMR) after 55. Disability-adjusted life years (DALYs) (Table 1; Figure 1D) peaked at 70–74 years, higher in males after 30. Globally (Table 2; Figures 2A–D), male/female patients, new cases, mortality cases, and DALYs peaked at 65–69/70–74 years, with males generally higher in most indicators after 40 and females overtaking after 90–95. From 1990 to 2021 (Tables 1, 2, 3, 4; Figures 3, 4), China's ASPR and ASIR increased, while ASMR and age-standardized DALY rate (ASDR) decreased; global ASPR/ASIR stabilized, and ASMR/ASDR declined, indicating similarities and differences in CVD burden.

**Table 1:** All-age cases and age-standardized prevalence rates, incidence rates, mortality rates, and DALY rates in China in 2021.

All-ages cases			Age-standardized rates per 100 000 people		
Measure	Sex	Val (95%UI)	Measure	Sex	Val (95%UI)
Prevalence	Male	64,714,213 (59,455,120, 70,456,527)	Prevalence	Male	6,616.82 (6,114.05, 7,152.17)
Prevalence	Female	69,131,342 (63,898,712, 74,538,436)	Prevalence	Female	6,587.69 (6,121.03, 7,073.13)
Prevalence	Both	133,845,555 (123,702,971, 144,518,968)	Prevalence	Both	6,603.72 (6,121.9, 7,087.64)
Incidence	Male	8,011,976 (7,159,131, 8,929,069)	Incidence	Male	847.06 (763.94, 932.68)
Incidence	Female	8,027,178 (7,229,384, 8,880,298)	Incidence	Female	772.86 (702.66, 850.06)
Incidence	Both	16,039,153 (14,443,325, 17,780,115)	Incidence	Both	811.81 (736.14, 892.01)
Deaths	Male	2,859,988 (2,320,770, 3,510,872)	Deaths	Male	372.54 (308.34, 447.92)
Deaths	Female	2,225,470 (1,771,802, 2,722,601)	Deaths	Female	217.02 (170.77, 264.96)
Deaths	Both	5,085,458 (4,311,063, 5,904,298)	Deaths	Both	280.11 (237.9, 323.9)
DALYs	Male	59,385,520 (47,906,695, 73,026,963)	DALYs	Male	6,637.72 (5,439.58, 8,038.8)
DALYs	Female	40,823,215 (33,020,456, 49,137,763)	DALYs	Female	3,877.91 (3,140.03, 4,655.37)
DALYs	Both	100,208,734 (84,648,255, 116,556,037)	DALYs	Both	5,120.06 (4,340.16, 5,935.79)

**Table 2:** All-age cases and age-standardized prevalence rates, incidence rates, mortality rates, and DALY rates in the world in 2021.

All-ages cases			Age-standardized rates per 100 000 people		
Measure	Sex	Val (95%UI)	Measure	Sex	Val (95%UI)
Prevalence	Male	307,987,850 (286,991,630, 328,533,218)	Prevalence	Male	7,666.88 (7,159.91, 8,166.57)
Prevalence	Female	304,071,472 (283,988,206, 322,690,548)	Prevalence	Female	6,750.55 (6,298.73, 7,163.16)
Prevalence	Both	612,059,322 (570,319,528, 649,806,889)	Prevalence	Both	7,178.73 (6,696.15, 7,620.67)
Incidence	Male	34,556,598 (31,400,608, 38,006,727)	Incidence	Male	866.76 (788.48, 950.01)
Incidence	Female	32,253,239 (29,554,892, 35,341,563)	Incidence	Female	712.96 (654.61, 777.9)
Incidence	Both	66,809,837 (60,907,604, 73,102,151)	Incidence	Both	787.04 (719.74, 859.7)
Deaths	Male	10,237,143 (9,521,531, 10,974,276)	Deaths	Male	281.11 (259.77, 301.13)
Deaths	Female	9,177,710 (8,159,423, 9,953,010)	Deaths	Female	196.69 (175.27, 213.19)
Deaths	Both	19,414,853 (17,775,807, 20,668,512)	Deaths	Both	235.18 (214.64, 250.52)
DALYs	Male	243,849,788 (228,038,411, 261,554,331)	DALYs	Male	6,169.02 (5,774.31, 6613.76)
DALYs	Female	184,477,625 (169,316,254, 197,156,951)	DALYs	Female	4,048.22 (3,721.38, 4,326.99)
DALYs	Both	428,327,412 (403,683,550, 453,711,663)	DALYs	Both	5,055.86 (4,759.55, 5,359.16)

**Table 3:** All-age cases and age-standardized prevalence rates, incidence rates, mortality rates, and DALY rates in China in 1990.

All-ages cases			Age-standardized rates per 100,000 people		
Measure	Sex	Val (95%UI)	Measure	Sex	Val (95%UI)
Prevalence	Male	25,403,270 (23,372,242, 27,178,606)	Prevalence	Male	5,976.81 (5,535.16, 6,373.11)
Prevalence	Female	26,961,220 (24,935,412, 28,883,234)	Prevalence	Female	6,078.83 (5,658.66, 6,472.64)
Prevalence	Both	52,364,490 (48,335,272, 55,935,626)	Prevalence	Both	6,024.24 (5,599.03, 6,393.02)
Incidence	Male	3,110,217 (2,839,701, 3,416,268)	Incidence	Male	800.42 (725.25, 885.47)
Incidence	Female	3,138,374 (2,886,290, 3,429,489)	Incidence	Female	766.26 (703.46, 835.55)
Incidence	Both	6,248,590 (5,727,577, 6,853,739)	Incidence	Both	783.9 (718.42, 857.63)
Deaths	Male	1,263,676 (1,059,252, 1,467,420)	Deaths	Male	466.57 (402.95, 526.73)
Deaths	Female	1,233,386 (1,051,904, 1,432,148)	Deaths	Female	369.94 (314.36, 428.11)
Deaths	Both	2,497,062 (2,206,621, 2,780,395)	Deaths	Both	407.72 (361.4, 452.12)
DALYs	Male	33,624,415 (28,057,256, 39,142,923)	DALYs	Male	9,030.23 (7,686.99, 10,344.5)
DALYs	Female	29,629,418 (25,367,604, 34,469,800)	DALYs	Female	7,329.96 (6,305.58, 8,477.9)
DALYs	Both	63,253,834 (56,338,614, 70,379,371)	DALYs	Both	8,074.76 (7,162.99, 8,953.39)

**Table 4:** All-age cases and age-standardized prevalence rates, incidence rates, mortality rates, and DALY rates in the world in 1990.

All-ages cases			Age-standardized rates per 100 000 people		
Measure	Sex	Val (95%UI)	Measure	Sex	Val (95%UI)
Prevalence	Male	143,650,946 (134,329,282, 152,093,063)	Prevalence	Male	7,633.11 (7,155.82, 8,068.97)
Prevalence	Female	145,899,286 (136,689,777, 154,033,709)	Prevalence	Female	6,681.46 (6,271.32, 7,040.17)
Prevalence	Both	289,550,232 (270,659,431, 305,371,954)	Prevalence	Both	7,116.05 (6,668.14, 7,482.02)
Incidence	Male	17,815,951 (16,179,198, 19,589,078)	Incidence	Male	976.34 (886.44, 1,072.95)
Incidence	Female	16,926,400 (15,549,870, 18,422,505)	Incidence	Female	791.69 (727.55, 860.23)
Incidence	Both	34,742,351 (31,845,969, 37,984,786)	Incidence	Both	878.79 (808.17, 959.9)
Deaths	Male	6,083,876 (5,784,071, 6,360,988)	Deaths	Male	401.85 (379.07, 419.96)
Deaths	Female	6,246,133 (5,770,037, 6,589,927)	Deaths	Female	320.71 (293.54, 338.59)
Deaths	Both	12,330,009 (11,626,405, 12,787,472)	Deaths	Both	358.12 (333.74, 372.63)
DALYs	Male	160,008,671 (152,644,576, 167,435,315)	DALYs	Male	8,726.81 (8,320.22, 9,112.03)
DALYs	Female	137,498,637 (128,905,670, 144,601,936)	DALYs	Female	6,485.82 (6,070.33, 6,814.14)
DALYs	Both	297,507,308 (284,600,935, 309,345,618)	DALYs	Both	7,550.17 (7,181.46, 7,861.99)

### Joinpoint regression analysis

**Figure 5:**
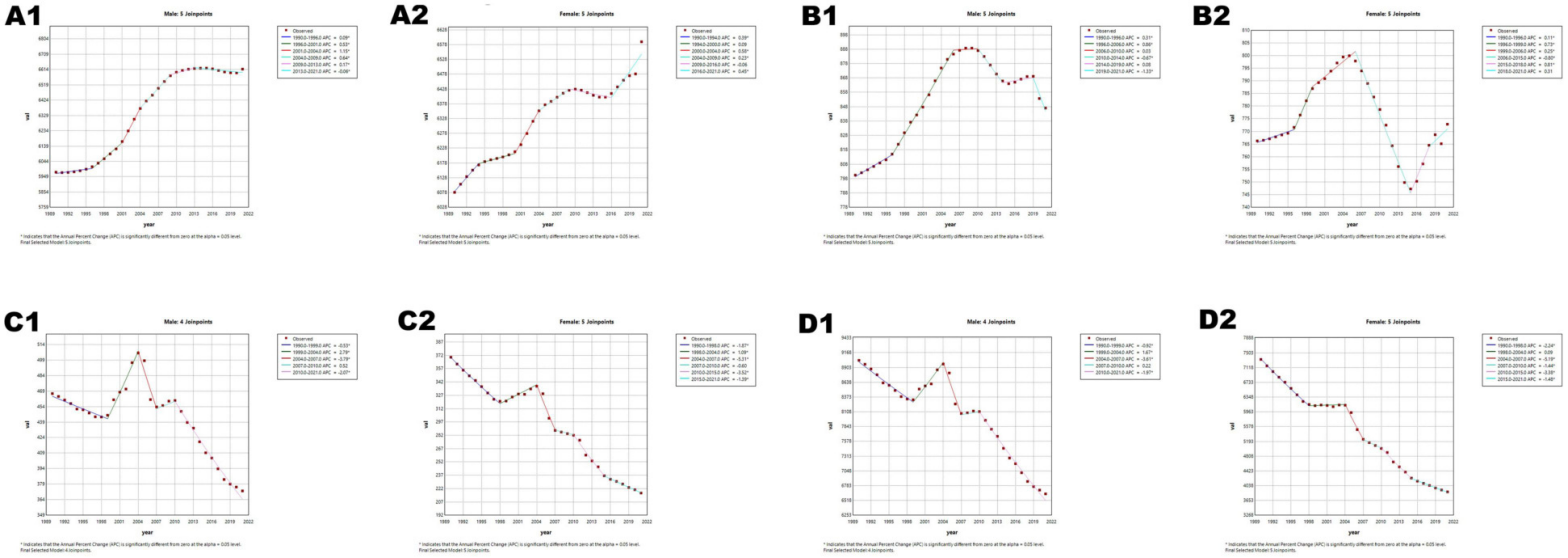
Joinpoint regression analysis of age-standardized rates (ASRs) for CVD in China and globally from 1990 to 2021. **(A)** Age-standardized incidence rate (ASIR). **(B)** Age-standardized prevalence rate (ASPR). **(C)** Age-standardized mortality rate (ASMR). **(D)** Age-standardized disability-adjusted life years rate (ASDR). APC* denoted significant *p*-values (<0.05), indicating statistically significant changes in APC. Figures A1–D1 represents males, and Figures A2–D2 represents females.

**Figure 6:**
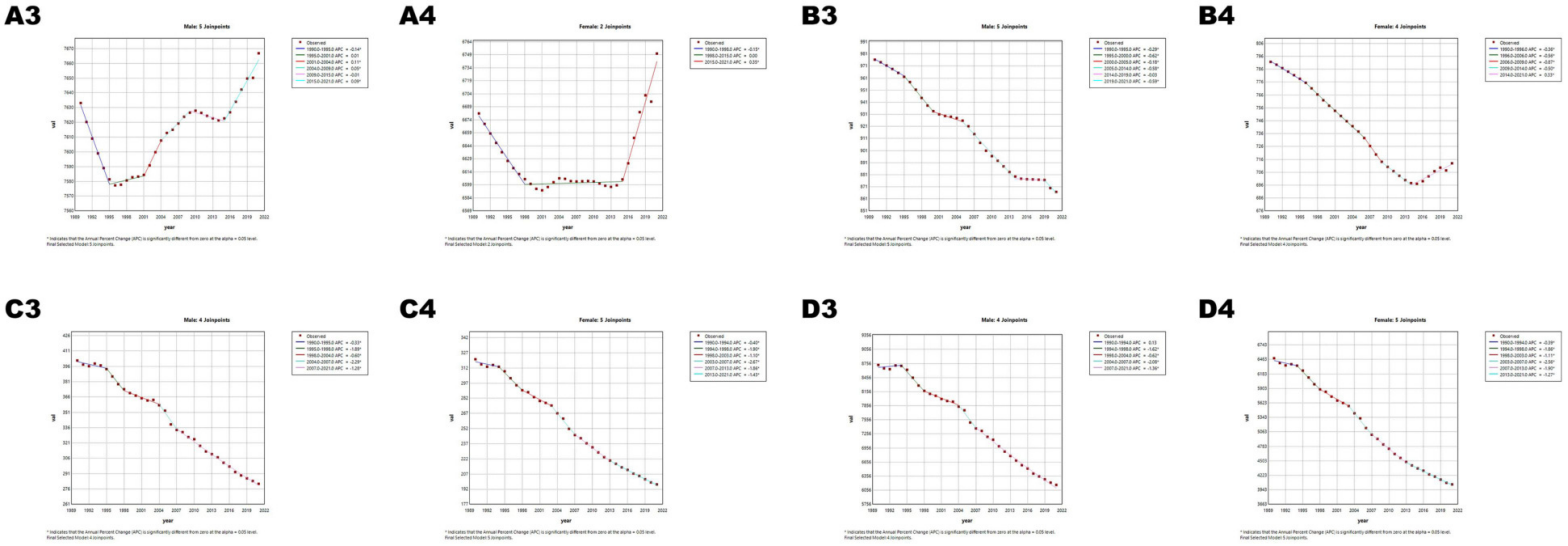
Joinpoint regression analysis of ASRs for CVD in global and globally from 1990 to 2021. **(A)** ASIR, **(B)** ASPR, **(C)** ASMR, **(D)** ASDR. APC* denoted significant *p*-values (<0.05), indicating statistically significant changes in APC. Figures A3–D3 represents males, and Figures A4–D4 represents females.

Age-standardized prevalence (ASPR), incidence (ASIR), mortality (ASMR), and disability-adjusted life year rates (ASDR) of CVD showed divergent trends in China and globally (Figures 5, 6; Tables 5, 6). China's ASPR (AAPC = 0.29) and ASIR (AAPC = 0.11) increased, exceeding global trends (0.03 and −0.35), with steeper rises in males. While China's ASMR (AAPC = −1.22) and ASDR (AAPC = −1.48) decreased, the decline rates were slower (ASMR) and faster (ASDR) than global levels (−1.35 and −1.27), with females showing greater declines. Notable changes in age-standardized rates (ASRs) occurred in Chinese males (2013–2021), Chinese females (2019–2021), and global females (2014–2021), warranting attention.

**Table 5:** Joinpoint regression analysis: trends in age-standardized prevalence rates, incidence rates, mortality rates, and DALYs (per 100,000 population) among both males and females in China from 1990 to 2019.

ASPR					ASIR					ASMR					ASDR				
Gender	Segment. Start	Segment. End	APC(95%CI)	AAPC (95% CI)	Gender	Segment. Start	Segment. End	APC (95%CI)	AAPC (95% CI)	Gender	Segment. Start	Segment. End	APC (95%CI)	AAPC (95% CI)	Gender	Segment. Start	Segment. End	APC (95%CI)	AAPC (95% CI)
Both	1990	2000	0.21 (0.20–0.23)	0.29 (0.27–0.31)	Both	1990	1995	0.17 (0.10–0.24)	0.11 (0.07–0.14)	Both	1990	1998	−1.32 (−1.49 to −1.15)	−1.22 (−1.42 to −1.02)	Both	1990	1998	−1.64 (−1.75 to −1.53)	−1.48 (−1.60 to −1.35)
Both	2000	2004	0.83 (0.74–0.91)		Both	1995	2005	0.64 (0.61–0.67)		Both	1998	2004	1.62 (1.31–1.92)		Both	1998	2004	0.69 (0.50–0.88)	
Both	2004	2009	0.45 (0.39–0.50)		Both	2005	2009	−0.13 (−0.29 to −0.03)		Both	2004	2007	−4.55 (−5.73 to −3.35)		Both	2004	2007	−4.27 (−5.01 to −3.52)	
Both	2009	2019	0.03 (0.01–0.04)		Both	2009	2015	−0.69 (−0.76 to −0.62)		Both	2007	2010	0.25 (−1.10 to −1.62)		Both	2007	2010	−0.39 (−1.23 to −0.44)	
Both	2019	2021	0.53 (0.35–0.71)		Both	2015	2019	0.48 (0.32–0.64)		Both	2010	2015	−2.82 (−3.30 to −2.33)		Both	2010	2016	−2.56 (−2.79 to −2.32)	
Female	1990	1994	0.39 (0.18–0.59)	0.24 (0.17–0.31)	Both	2019	2021	−0.61 (−0.96 to −0.26)		Both	2015	2021	−1.57 (−1.95 to −1.19)		Both	2016	2021	−1.42 (−1.76 to −1.07)	
Female	1994	2000	0.09 (−0.05 to −0.23)		Female	1990	1996	0.11 (0.00–0.22)	0.02 (−0.07 to −0.12)	Female	1990	1998	−1.87 (−2.07 to −1.67)	−1.70 (−1.93 to −1.47)	Female	1990	1998	−2.24 (−2.39 to −2.10)	−2.05 (−2.21 to −1.89)
Female	2000	2004	0.58 (0.26–0.90)		Female	1996	1999	0.73 (0.07–1.39)		Female	1998	2004	1.09 (0.74–1.44)		Female	1998	2004	0.09 (-0.16–0.34)	
Female	2004	2009	0.23 (0.03–0.43)		Female	1999	2006	0.25 (0.14–0.37)		Female	2004	2007	−5.31 (−6.62 - −3.98)		Female	2004	2007	−5.19 (-6.10 - -4.27)	
Female	2009	2016	−0.06 (−0.17 to −0.04)		Female	2006	2015	−0.80 (−0.87 to −0.73)		Female	2007	2010	−0.60 (−2.17 to −1.00)		Female	2007	2010	−1.44 (−2.50 to −0.37)	
Female	2016	2021	0.45 (0.30–0.61)		Female	2015	2018	0.81 (0.17–1.45)		Female	2010	2015	−3.52 (−4.07 to −2.95)		Female	2010	2015	−3.38 (−3.77 to −2.98)	
Male	1990	1996	0.09 (0.04–0.14)	0.32 (0.28–0.36)	Female	2018	2021	0.31 (-0.05–0.66)		Female	2015	2021	−1.39 (−1.84 to −0.95)		Female	2015	2021	−1.48 (−1.79 to −1.17)	
Male	1996	2001	0.53 (0.43–0.62)		Male	1990	1996	0.31 (0.25–0.38)	0.18 (0.13–0.23)	Male	1990	1999	−0.53 (−0.76 to −0.30)	−0.77 (−1.06 to −0.49)	Male	1990	1999	−0.92 (−1.08 to −0.76)	−1.04 (−1.22 to −0.85)
Male	2001	2004	1.15 (0.86–1.45)		Male	1996	2006	0.86 (0.82–0.90)		Male	1999	2004	2.79 (2.17–3.42)		Male	1999	2004	1.67 (1.24–2.09)	
Male	2004	2009	0.64 (0.54–0.73)		Male	2006	2010	0.03 (-0.17–0.22)		Male	2004	2007	−3.79 (−5.51 to −2.03)		Male	2004	2007	−3.61 (−4.77 to −2.43)	
Male	2009	2013	0.17 (0.02–0.31)		Male	2010	2014	−0.67 (−0.86 to −0.48)		Male	2007	2010	0.52 (−1.59 to −2.68)		Male	2007	2010	0.22 (−1.16 to −1.63)	
Male	2013	2021	−0.06 (−0.09 to −0.02)		Male	2014	2019	0.08 (−0.04 to −0.20)		Male	2010	2021	−2.07 (−2.30 to −1.85)		Male	2010	2021	−1.97 (−2.12 to −1.82)	
					Male	2019	2021	−1.33 (−1.73 to −0.93)											

**Table 6:** Joinpoint regression analysis: trends in age-standardized prevalence rates, incidence rates, mortality rates, and DALYs (per 100,000 population) among both males and females globally from 1990 to 2019.

ASPR					ASIR					ASMR					ASDR				
Gender	Segment. Start	Segment. End	APC (95%CI)	AAPC (95% CI)	Gender	Segment. Start	Segment. End	APC (95%CI)	AAPC (95% CI)	Gender	Segment. Start	Segment. End	APC (95%CI)	AAPC (95% CI)	Gender	Segment. Start	Segment. End	APC (95%CI)	AAPC (95% CI)
Both	1990	1997	−0.13 (−0.15 to −0.10)	0.03 (0.01–0.04)	Both	1990	1996	−0.32 (−0.40 to −0.24)	−0.35 (-0.40 to −0.30)	Both	1990	1994	−0.21 (−0.59 to −0.16)	−1.35 (−1.49 to −1.21)	Both	1990	1994	−0.07 (−0.34 to −0.21)	−1.27 (−1.37 to −1.17)
Both	1997	2008	0.04 (0.02–0.05)		Both	1996	2000	−0.63 (−0.86 to −0.40)		Both	1994	1998	−1.73 (−2.28 to −1.17)		Both	1994	1998	−1.70 (−2.09 to −1.31)	
Both	2008	2015	0.00 (−0.03 to −0.03)		Both	2000	2005	−0.31 (−0.46 to −0.16)		Both	1998	2004	−0.94 (−1.19 to −0.69)		Both	1998	2004	−0.91 (−1.07 to −0.74)	
Both	2015	2021	0.22 (0.18–0.25)		Both	2005	2009	−0.76 (−0.98 to −0.54)		Both	2004	2007	−2.72 (−3.83 to −1.60)		Both	2004	2007	−2.40 (−3.16 to −1.63)	
Female	1990	1998	−0.15 (−0.18 to −0.12)	0.03 (0.02–0.04)	Both	2009	2014	−0.49 (−0.62 to −0.36)		Both	2007	2021	−1.44 (−1.51 to −1.38)		Both	2007	2015	−1.59 (−1.70 to −1.47)	
Female	1998	2015	0.00 (−0.01 to −0.01)		Both	2014	2021	0.08 (0.02–0.14)		Female	1990	1994	−0.40 (−0.77 to −0.02)	−1.55 (−1.68 to −1.42)	Both	2015	2021	−1.16 (−1.34 to −0.97)	
Female	2015	2021	0.35 (0.30–0.40)		Female	1990	1996	−0.36 (−0.42 to −0.29)	−0.34 (−0.39 to −0.30)	Female	1994	1998	−1.90 (−2.46 to −1.34)		Female	1990	1994	−0.39 (−0.73 to −0.05)	−1.50 (−1.61 to −1.38)
Male	1990	1995	−0.14 (−0.16 to −0.12)	0.01 (0.00–0.02)	Female	1996	2006	−0.56 (−0.60 to −0.53)		Female	1998	2003	−1.10 (−1.45 to −0.75)		Female	1994	1998	−1.86 (−2.32 to −1.39)	
Male	1995	2001	0.01 (−0.00–0.03)		Female	2006	2009	−0.87 (−1.26 to −0.48)		Female	2003	2007	−2.67 (−3.23 to −2.10)		Female	1998	2003	−1.11 (−1.42 to −0.80)	
Male	2001	2004	0.11 (0.04–0.17)		Female	2009	2014	−0.50 (−0.62 to −0.38)		Female	2007	2013	−1.86 (−2.14 to −1.58)		Female	2003	2007	−2.56 (−3.03 to −2.08)	
Male	2004	2009	0.05 (0.03–0.07)		Female	2014	2021	0.33 (0.27–0.38)		Female	2013	2021	−1.43 (−1.59 to −1.27)		Female	2007	2013	−1.90 (−2.13 to −1.66)	
Male	2009	2015	−0.01 (−0.03 to −0.00)		Male	1990	1995	−0.29 (−0.37 to −0.21)	−0.39 (−0.42 to −0.35)	Male	1990	1995	−0.33 (−0.56 to −0.10)	−1.15 (−1.29 to −1.02)	Female	2013	2021	−1.27 (−1.41 to −1.13)	
Male	2015	2021	0.09 (0.08–0.10)		Male	1995	2000	−0.62 (−0.74 to −0.51)		Male	1995	1998	−1.89 (−2.83 to −0.94)		Male	1990	1994	0.13 (-0.24–0.51)	−1.13 (−1.25 to −1.00)
					Male	2000	2005	−0.18 (−0.29 to −0.07)		Male	1998	2004	−0.60 (−0.81 to −0.40)		Male	1994	1998	−1.62 (−2.13 to −1.11)	
					Male	2005	2014	−0.58 (−0.62 to −0.55)		Male	2004	2007	−2.29 (−3.20 to −1.37)		Male	1998	2004	−0.62 (−0.84 to −0.40)	
					Male	2014	2019	−0.03 (−0.13 to 0.07)		Male	2007	2021	−1.28 (−1.34 to −1.22)		Male	2004	2007	−2.08 (−3.04 to −1.12)	
					Male	2019	2021	−0.59 (−0.94 to −0.25)							Male	2007	2021	−1.36 (−1.42 to −1.29)	

### Age-period-cohort analysis

**Figure 7:**
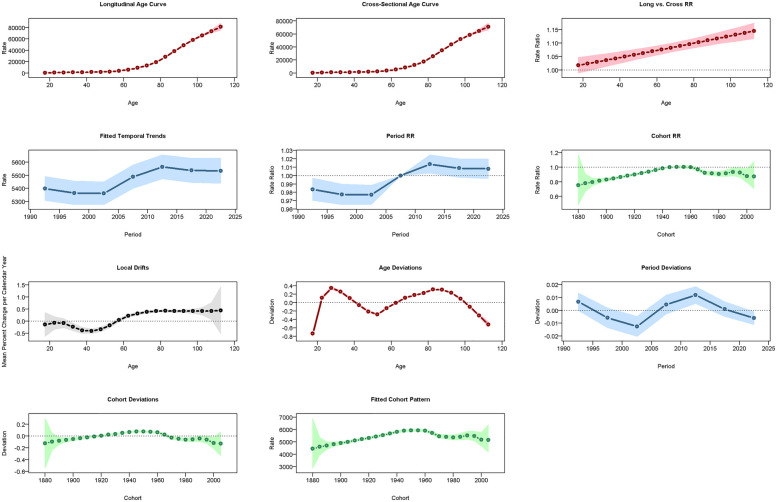
Results of the age-period-cohort model (APCM) for the prevalence of CVD in China. RR: relative risk.

**Figure 8:**
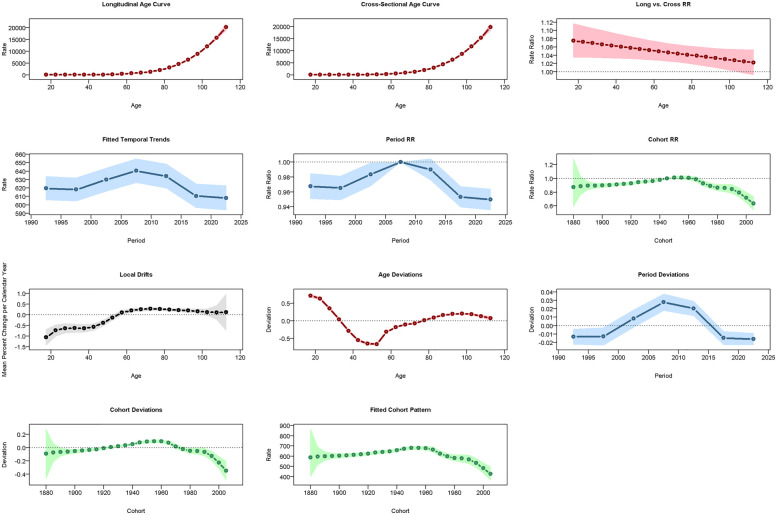
Results of the APCM for the incidence of CVD in China.

**Figure 9:**
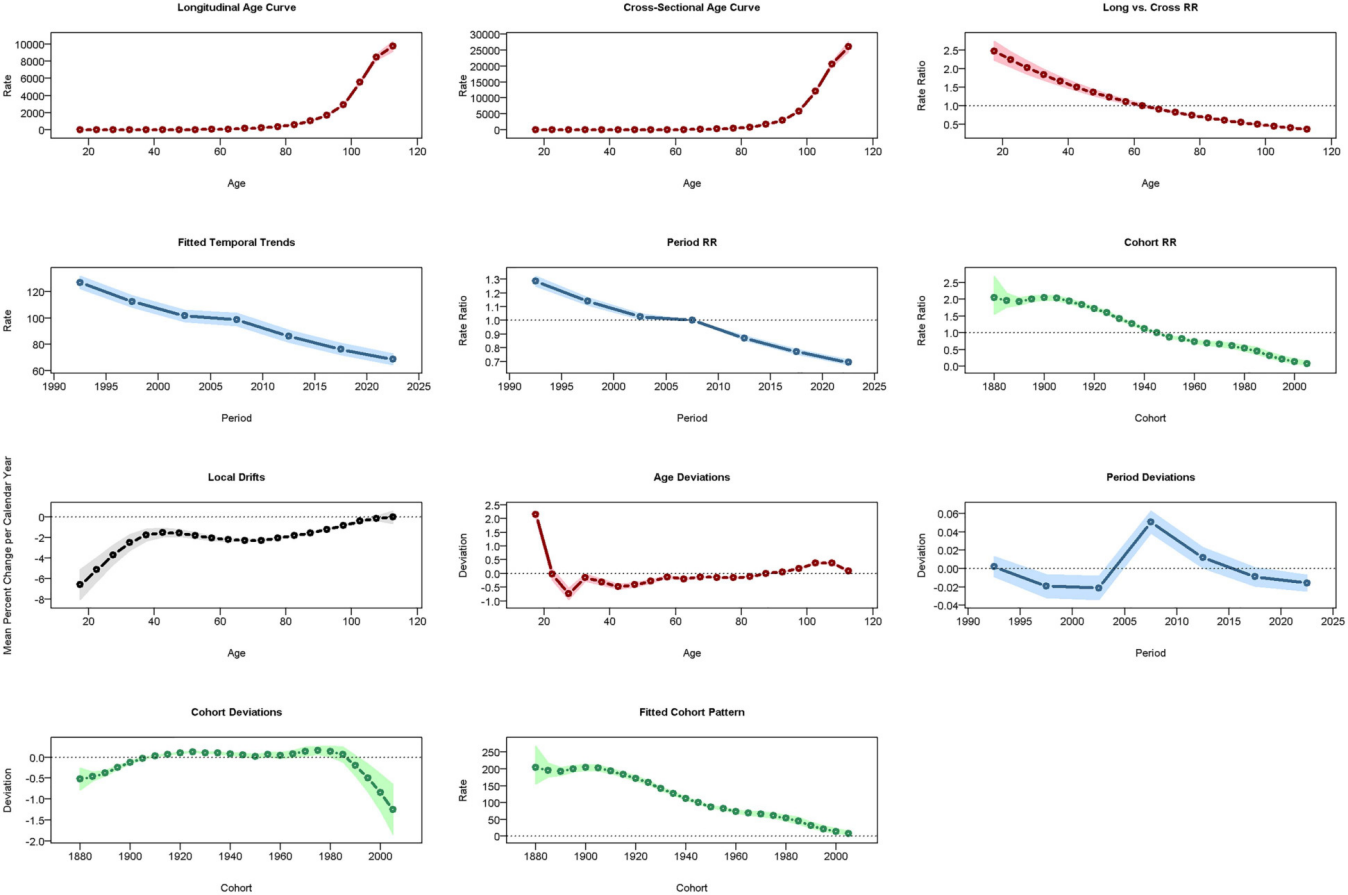
Results of the APCM for the mortality of CVD in China.

In China, CVD rates increased with age: ASPR rose vs. 2005–2010 but fell vs. 1960 (Figure 7), ASIR declined vs. both (Figure 8), and ASMR/ASDR dropped vs. 2005–2010 and 1940 (Figure 9; Supplementary Figure 1). Globally, ASPR increased vs. 2005–2010 and 1920–1940 (Supplementary Figure 2), ASIR fell vs. 2005–2010 but rose vs. 1940–1980 (Supplementary Figure 3), and ASMR/ASDR decreased vs. both periods (Supplementary Figures 4, 5).

### Decomposition analysis

Decomposition analysis (Supplementary Figures 6, 7, Supplementary Tables 1, 2) showed that increases in China's CVD prevalence/incidence were driven by population aging/growth, while mortality/DALYs rates were driven by aging/epidemiological trends. Globally, prevalence was driven by aging/population growth, incidence/mortality/DALYs by population growth/epidemiological trends, aging/epidemiological trends, and population growth/epidemiological trends, respectively. Epidemiological trends negatively impacted mortality and DALYs but did not alter overall trends. Notably, these factors can interact and potentially amplify each other: for instance, rapid population aging may exacerbate the impact of epidemiological trends, such as rising metabolic risk factors, leading to higher disease burden. Understanding these synergistic effects is critical for planning effective CVD prevention strategies in the context of China's rapidly aging population.

### Predictions for the prevalence, incidence, mortality, and DALYs of CVD over the next 15 years (using ARIMA)

ARIMA model (Supplementary Figures 8, 9) projects China's CVD ASPR to rise, ASIR/ASMR stable, ASDR declining over 15 years; global ASPR/ASIR/ASMR stable, ASDR decreasing. However, these long-term forecasts carry inherent uncertainty, particularly due to potential changes in risk factor prevalence, healthcare policies, and technological advancements. Future research could explore scenario-based modeling with varying assumptions to assess the robustness of these predictions.

### Predictions for the prevalence, incidence, mortality, and DALYs of CVD over the next 15 years (using BAPC)

Using 1990–2021 GBD data, a BAPC model predicted 2022–2036 CVD burden (Supplementary Figures 10, 11): ASPR up in both sexes (females more), ASIR down in men/up in women, ASMR/ASDR down globally and in China.

### Cross-Validation and predictive accuracy

For the ARIMA model (Supplementary Table 3), relative errors for prevalence, incidence, mortality, and DALYs were generally low (<7% for all indicators except mortality in Chinese males, which reached 6.76%), indicating good predictive performance. Although some R^2^ values were negative (e.g., −0.9471 for Chinese male prevalence, −66.4149 for Chinese male deaths), this is a known limitation of R^2^ for ARIMA time series models, as it may underestimate model performance when dynamic trends dominate static variance. Importantly, RMSE and MAE values remained reasonably small, suggesting that absolute prediction errors were minimal and model forecasts are robust.

For the BAPC model (Supplementary Table 4), *R*^2^ values were uniformly very high (>0.999), with relative errors consistently below 1% for most indicators, and RMSE/MAE within acceptable ranges, demonstrating excellent model fit and predictive reliability.

Overall, the combination of ARIMA and BAPC results provides strong evidence for the robustness of our 15-year projections. The consistent trends across two modeling approaches, along with low relative errors and reasonable RMSE/MAE values, support the credibility of our forecasts, while acknowledging the inherent uncertainties associated with long-term predictions.

## Discussion

We utilized the 2021 GBD (Global Burden of Diseases) data to assess the overall CVD burden in China and globally from 1990 to 2021.

In 1990, the numbers of prevalent cases, new cases, mortality cases, and disability-adjusted life years (DALYs) of cardiovascular diseases (CVDs) globally were 437.7%, 459.5%, 394.2%, and 369.7% higher than those in China, respectively. The age-standardized prevalence rate (ASPR) and age-standardized incidence rate (ASIR) globally were 18.1% and 12.1% higher than those in China. However, the age-standardized mortality rate (ASMR) and age-standardized disability-adjusted life year rate (ASDR) in China were 13.8% and 6.9% higher than the global levels, respectively. When analyzed by gender, the values of multiple indicators for both global males and females far exceeded those of China. Only some data of ASMR and ASDR in China were higher than the global values. It can be seen that the burden of CVDs globally was heavier in 1990. Although the scope of influence in China was relatively smaller, the death risk and health loss of patients were higher. In 2021, the numbers of prevalent cases, new cases, mortality cases, and DALYs of CVDs globally were 356.77%, 316.65%, 371.77%, and 327.42% higher than those in China, respectively. The ASPR globally was 8.71% higher than that in China, while the ASIR, ASMR, and ASDR in China were 3.14%, 19.10%, and 1.27% higher than the global levels, respectively. After analyzing by gender, most of the indicators globally were higher than those in China. This indicates that the overall burden of CVDs globally was still heavy in 2021. The incidence rate and mortality rate in China were relatively high, and the burden of disability-adjusted life years caused by the disease was slightly higher than the global average. When comparing the data of 1990 and 2021, the increasing amplitudes of global indicators such as the number of prevalent cases relative to those in China decreased, indicating an improvement in China's medical and health service capacity. However, the relative increases in ASIR and ASMR and the relative decrease in ASDR in China suggest that the incidence rate and mortality rate were relatively high, while the disease burden was relatively reduced. Recent evidence indicates that this slower decline in CVD mortality may be partly due to uneven access to primary healthcare and suboptimal hypertension control, which similarly affects stroke management and likely extends to broader CVD management. Significant regional disparities in stroke burden exist across China, with provinces having better primary healthcare accessibility and higher hypertension control rates experiencing lower cerebrovascular mortality, underscoring the crucial role of equitable healthcare distribution in reducing cardiovascular burden. China still needs to strengthen the construction of the medical and health system, considering potential regional disparities in healthcare access and CVD management. Over the past three decades, several nationwide health reforms and public health initiatives have contributed to the relatively faster decline in age-standardized mortality and DALY rates from CVDs in China compared with the global average. Beginning with the launch of the New Rural Cooperative Medical Scheme (NRCMS) in 2003 and the Urban Resident Basic Medical Insurance in 2007, China achieved near-universal health insurance coverage by the mid-2010s, greatly improving access to basic medical services, especially in rural areas. The Essential Drug System (2009) and the National Basic Public Health Service Program (NBPHSP) expanded the availability of affordable essential antihypertensive and lipid-lowering medications, which have been shown to reduce cardiovascular mortality. In addition, the establishment of regional emergency care networks for acute myocardial infarction and stroke—supported by the China Chest Pain Center and Stroke Center alliances—has significantly improved early diagnosis and treatment capacity, particularly through the “Green Channel” system for rapid patient triage and transfer. Broader social changes, including rapid urbanization accompanied by improved health literacy, better nutritional status, and the nationwide promotion of cardiovascular health guidelines (e.g., the Healthy China 2030 initiative), have also played a role. Together, these policy-driven transformations have likely accelerated the decline in CVD mortality and DALY rates beyond what would be expected from medical technology alone. Different provinces may face unique challenges and opportunities, and national averages may obscure these local variations, suggesting the importance of region-specific strategies (11).

Additionally, the inclusion of model validation results strengthens confidence in our 15-year CVD predictions. Cross-validation demonstrates that ARIMA predictions, despite some negative R^2^ values (e.g., −0.9471 for Chinese male prevalence and −66.4149 for Chinese male deaths), maintain low absolute errors (RMSE and MAE), indicating that the model accurately captures dynamic trends despite limitations of *R*^2^ for time series data. BAPC predictions show near-perfect *R*^2^ values (>0.999) and minimal prediction errors, reflecting excellent model fit. Together, these findings support the robustness of our long-term projections and suggest that the forecasts are reliable for informing public health planning and policy development, while acknowledging inherent uncertainties such as changes in risk factor prevalence and healthcare interventions.

Between 1990 and 2021, males generally bear a heavier burden of cardiovascular diseases (CVDs), which may be influenced by biological, behavioral, and social factors. The persistence of higher CVD burden among males likely reflects complex interactions between these determinants. Biologically, sex hormones such as estrogen may provide some vascular protection in premenopausal women, while postmenopausal estrogen decline may contribute to increased CVD risk in older women. Behaviorally, men often exhibit higher rates of smoking, alcohol consumption, and occupational stress, particularly in urban and industrial settings. Occupational exposures—such as long working hours, shift work, or high-temperature and polluted environments—may further elevate cardiovascular risk. Rapid urbanization and dietary changes, including increased intake of sodium, saturated fats, and processed foods, may introduce additional risks across both sexes. Over time, as female smoking and obesity rates rise and access to healthcare improves, the gender gap in CVD burden may narrow, highlighting the need for continuous monitoring. Differences in the effectiveness of preventive and therapeutic interventions between sexes suggest the potential value of sex-specific public health strategies. Future research could further explore how demographic transitions, urbanization, and dietary shifts specifically impact CVD patterns in men and women, as well as conduct cross-country comparisons with other rapidly developing nations to better understand sex-specific risk factors and inform targeted interventions. Although these data primarily focus on the comparison between global and Chinese contexts, gender differences are often a significant consideration in the health domain. By comparing data between males and females, we find that most indicators are higher among males than females, potentially indicating that males are more prominently affected by cardiovascular health issues. Factors such as females' physiological structure, physiological changes, and lifestyles may be involved and require further research (12, 13).

DALYs is a comprehensive health indicator commonly used to assess the total health loss due to diseases, injuries, and premature deaths in a population. It is one of the most widely used methods to measure health loss in GBD (Global Burden of Disease) studies, providing crucial insights for policymakers to understand the impact of various diseases, health conditions, or interventions on the overall population's health. This suggests that the years of life lost due to disability may be an issue of focus in the future. Based on current findings, these results largely indicate that the increase in the prevalence of CVDs globally and in China, the increase in incidence rates in China, and the increase in the number of incident cases globally may be related to demographic changes, such as the extension of life expectancy and population aging (14). The reduction in standardized mortality and DALYs rates is associated with improvements in healthcare, advancements in medical technology, and the widespread application of CVD screening programs. Additionally, the attributable burden of CVD risk factors suggests that effective management of these factors can prevent CVD. Overall, there is a significant downward trend in future mortality and DALYs rates for CVDs. However, targeted and effective strategies are still needed to prevent and treat CVDs.

Identifying key risk factors is essential for effectively reducing the prevalence and incidence of CVDs. In China, hypertension continues to be highly prevalent, despite general improvements in awareness and management, while elevated cholesterol levels have gradually increased, and smoking rates among men remain high. Globally, the prevalence of obesity and diabetes has also been rising, further contributing to the CVD burden. Temporal trends suggest that improvements in the management of these risk factors—such as better blood pressure control and reductions in tobacco exposure—are associated with decreases in age-standardized mortality and disease burden. Monitoring these trends over time can provide actionable insights for designing targeted interventions and public health strategies to mitigate cardiovascular risk.

Risk factor detection is crucial for the prevention of CVDs, and accurately locating risk factors is key to preventing them (15). The primary contributing factors to CVD-related deaths include metabolic, behavioral, and dietary risks. Hypertension, air pollution, high LDL-C, and smoking are also common risk factors (16). A report on cardiovascular health and disease in China categorizes factors affecting cardiovascular health into five aspects: tobacco, diet, physical activity, body weight, and mental health (6). BMI is a commonly used indicator to assess overweight and obesity, and excess body fat resulting from weight is considered a risk factor for many health issues, including CVDs. Over the past few decades, the global prevalence of obesity has increased. In 2013, some countries in Oceania, North Africa, and the Middle East had adult obesity rates exceeding 50%. High BMI can promote the occurrence and progression of CVDs (such as atrial fibrillation) through various mechanisms (17–19). An open-label, cluster-randomized trial (Salt Substitution and Stroke Study, SSaSS) showed that salt substitution can reduce the risk of cardiovascular events. A modeling study in China predicts that salt substitution could prevent 461,000 cardiovascular deaths and 743,000 non-fatal cardiovascular events annually. Salt substitution is the only cost-effective salt reduction intervention with Level 1 evidence and should be considered by all countries planning or implementing early prevention of CVDs (20–22). An important finding is that women face an increased risk of death from tobacco and sugary drinks, while men face a decreased risk. However, the attributable risk of secondhand smoke among women has significantly declined, indicating improved smoking control in public places. Efforts to control tobacco use should remain a priority in public health policies (23). Policymakers need to develop tobacco control and diet management plans for specific populations. Another way to reduce the burden of CVDs is to avoid exposure to high levels of air pollution (24–26). Air quality has significantly improved, with household air pollution from solid fuels decreasing from 19.14% to 4.52%, a drop of 76.38%. In contrast, environmental particulate pollution increased from 8.19% to 18.36%, an increase of 124.13%, indicating that outdoor air pollution's impact on CVD mortality is growing. The widespread use of clean energy may further reduce air pollution (27, 28). According to China's Ministry of Ecology and Environment, China's average particulate matter (PM2.5) concentration decreased by 57%, and the number of heavily polluted days decreased by 92% from 2013 to 2022. Furthermore, educating the general population about other risk factors can help improve the prevention of CVDs and reduce their burden.

Future efforts should focus on identifying relevant risk factors in patients with CVDs or those at high risk of CVDs and formulating reasonable health policies. Emphasis should be placed on developing new prevention strategies to alleviate the increasing burden of CVDs (29). Moreover, integrating basic research with clinical practice and developing new, naturally effective drugs can help reduce disparities in disease management and improve patient outcomes. Cross-disciplinary collaboration is critical in this context. Initiatives such as the China Cardiovascular Health Alliance exemplify how integrating epidemiology, clinical medicine, public health policy, and community-based interventions can achieve measurable reductions in CVD burden (16). Such collaborations facilitate the identification of high-risk populations, the design of context-specific interventions, the optimization of resource allocation, and the translation of research findings into actionable public health strategies. Promoting continuous research and fostering interdisciplinary networks can further strengthen CVD prevention and control, ultimately enhancing global cardiovascular health and well-being.

## Limitations

Our study has certain limitations. Firstly, the research did not categorize cardiovascular diseases into major subtypes such as ischemic heart disease and stroke. Since different CVD subtypes have distinct risk factors, pathophysiological mechanisms, and intervention strategies, the lack of such stratified analysis may restrict the clinical relevance and policy utility of our findings to a certain extent. We acknowledge this as an important consideration, and further studies are needed in the future to classify specific types of CVDs to assess their severity and trends in the study population. Secondly, there is a need to incorporate specific provincial research data and include more representative data from various regions to comprehensively understand the burden of CVDs. Additionally, a major limitation is the lack of sensitivity analysis to assess how changes in prior assumptions and observed data would affect the predictions of ARIMA and BAPC models. This increases the uncertainty of the results, suggesting that the interpretations should be approached with caution. Given the predictive nature of this study, sensitivity analyses representing reasonable scenarios are necessary to validate the robustness of the ARIMA and BAPC models. Future research could improve prediction accuracy by utilizing long-term case registry data or conducting regular sampling surveys. Sensitivity analyses and new data-based methods will provide more reliable predictions and help identify key factors influencing CVD trends. Nevertheless, this study provides valuable and updated findings regarding the burden of CVDs in China and globally.

## Conclusion

This study highlights the changes in the burden of CVDs in China over the past three decades, which stands in stark contrast to global trends. Despite the decline in age-standardized DALYs rates and mortality rates for CVDs in both China and globally from 1990 to 2021, the increase in prevalence rates of CVDs in both regions, the rise in incidence rates in China, and the growth in the number of cases globally indicate that the burden of CVDs remains high both in China and worldwide. Furthermore, overall, the burden of CVDs is higher among males than females. Given the large and rapidly aging populations in both China and globally, the burden of CVDs poses a major issue. There is an urgent need to develop and implement targeted and effective strategies and interventions, including evidence-based screening programs, lifestyle modification initiatives, tobacco and diet management policies, and healthcare system reforms, to alleviate the burden of CVDs and provide actionable guidance for policymakers.

Furthermore, promoting interdisciplinary collaboration across epidemiology, clinical medicine, public health, and policy-making is essential to enhance the effectiveness of CVD research, prevention, and intervention strategies.

## Data Availability

Publicly available datasets were analyzed in this study. This data can be found here: https://vizhub.healthdata.org/gbd-results/.
