# Supplementary figures and images for: Systematic analysis and comparison of the burden of cardiovascular diseases in China from 1990 to 2021 and its 15-year prediction with global levels
Source: Front Cardiovasc Med. 2025 Dec 4;12:1660259. doi: 10.3389/fcvm.2025.1660259 (PMC12711838; doi:10.3389/fcvm.2025.1660259)

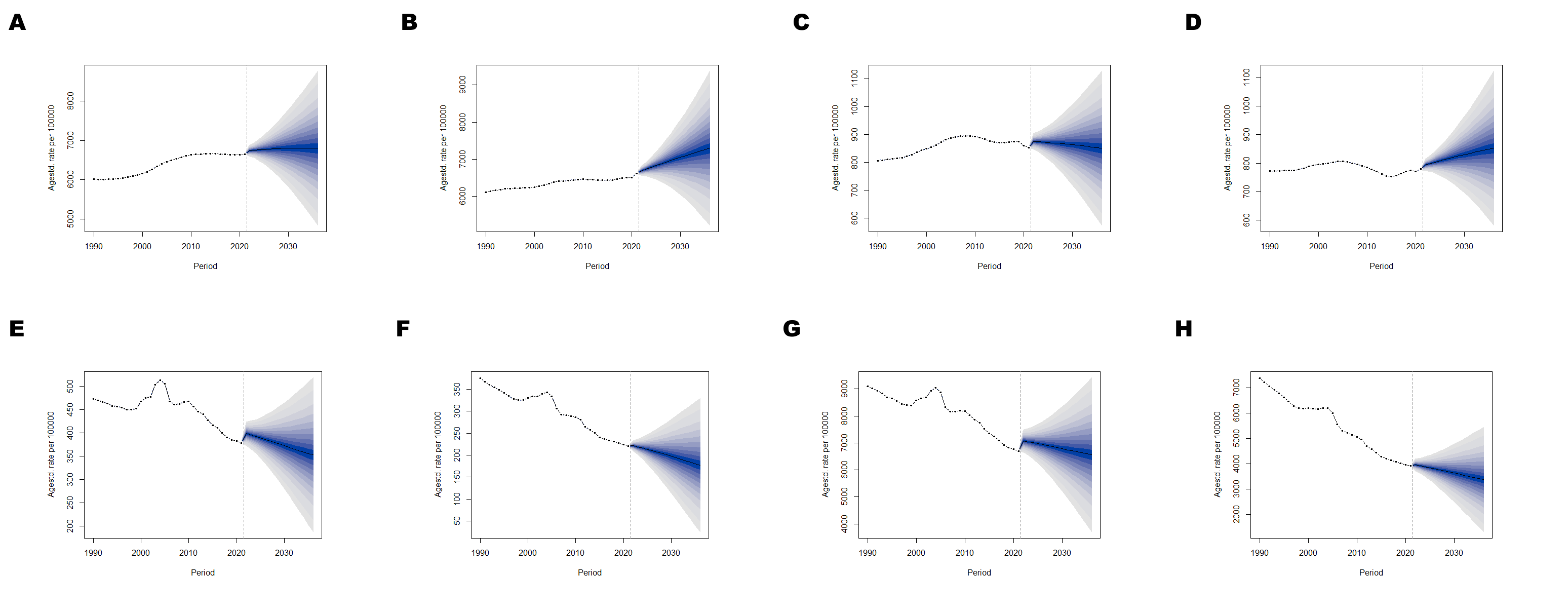

Supplement: Supplementary file 1 [file Datasheet1.zip › Supplementary Materials_Revised_2/Figure S10/Figure S10 A-H.png]

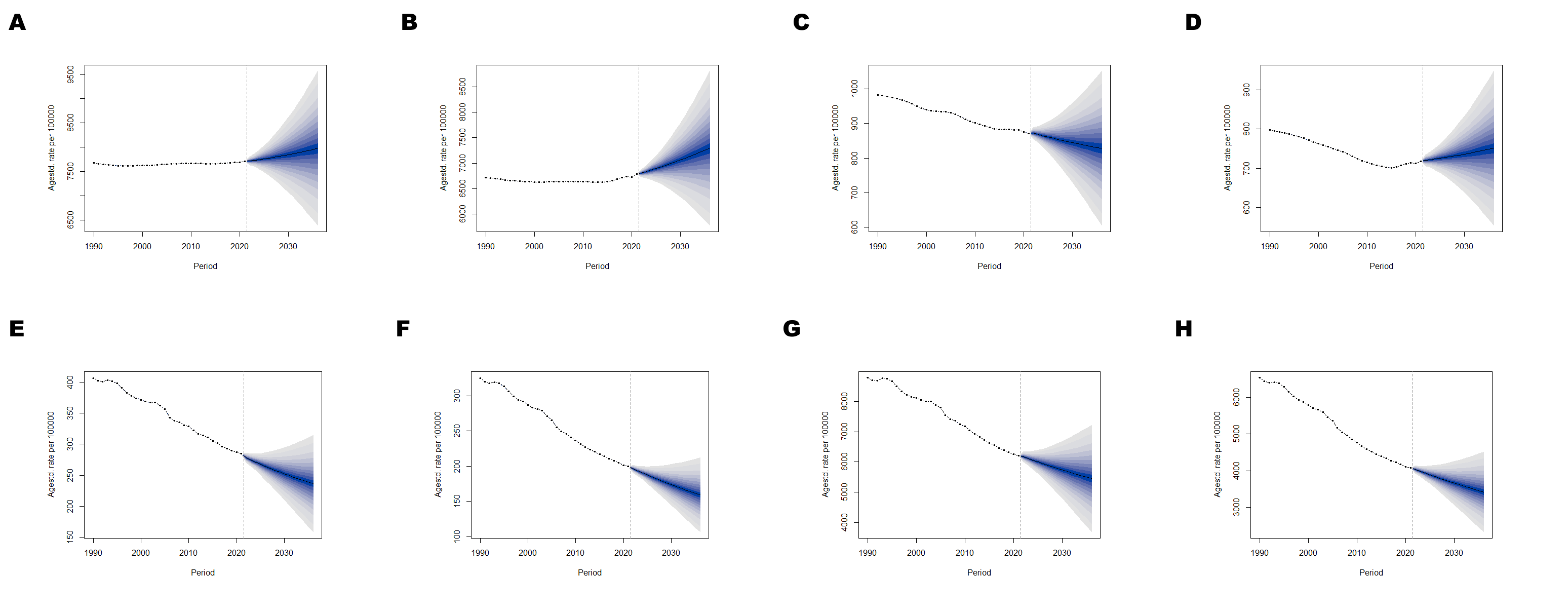

Supplement: Supplementary file 1 [file Datasheet1.zip › Supplementary Materials_Revised_2/Figure S11/Figure S11 A-H.png]

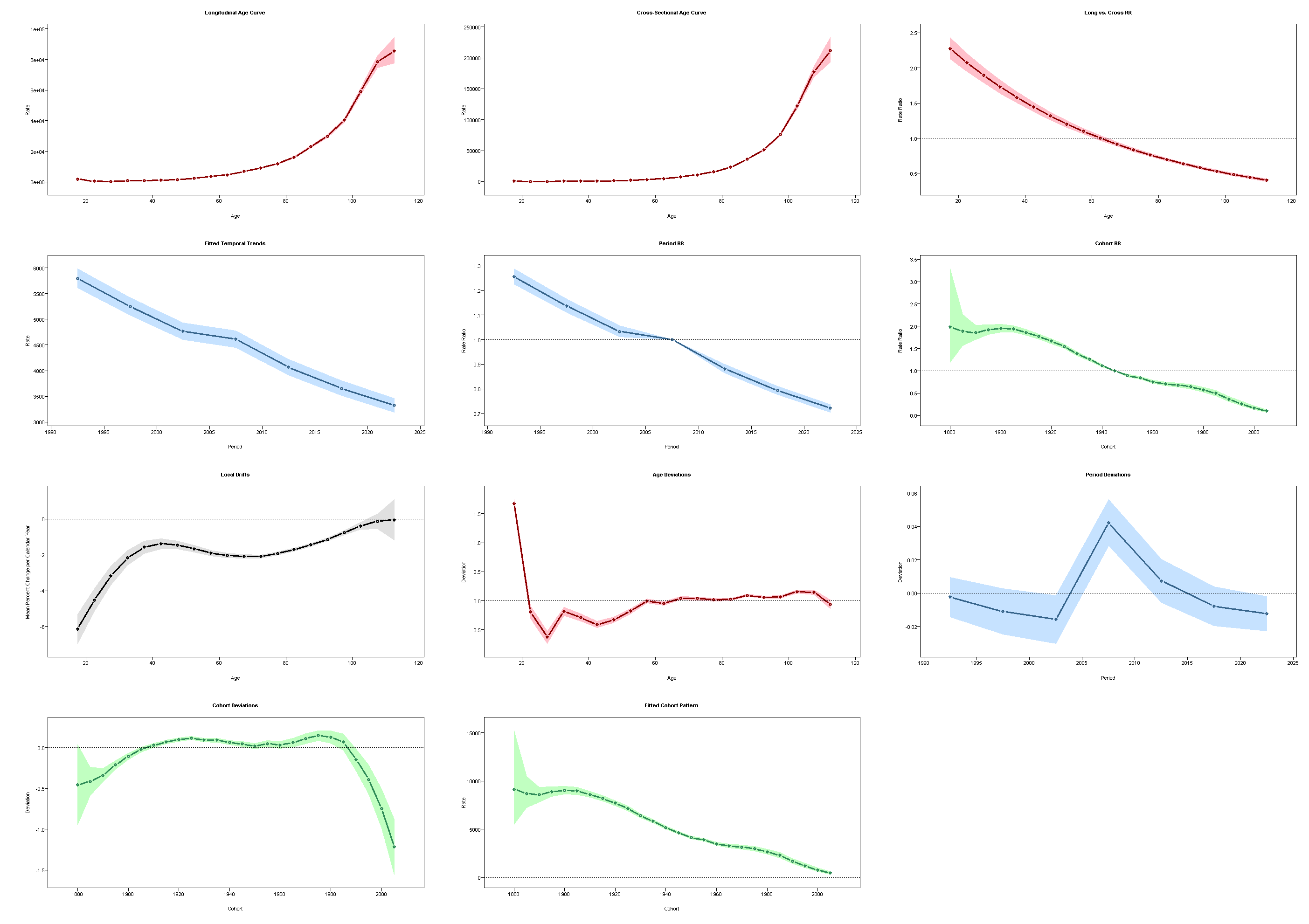

Supplement: Supplementary file 1 [file Datasheet1.zip › Supplementary Materials_Revised_2/Figure S1-S5/Figure S1.png]

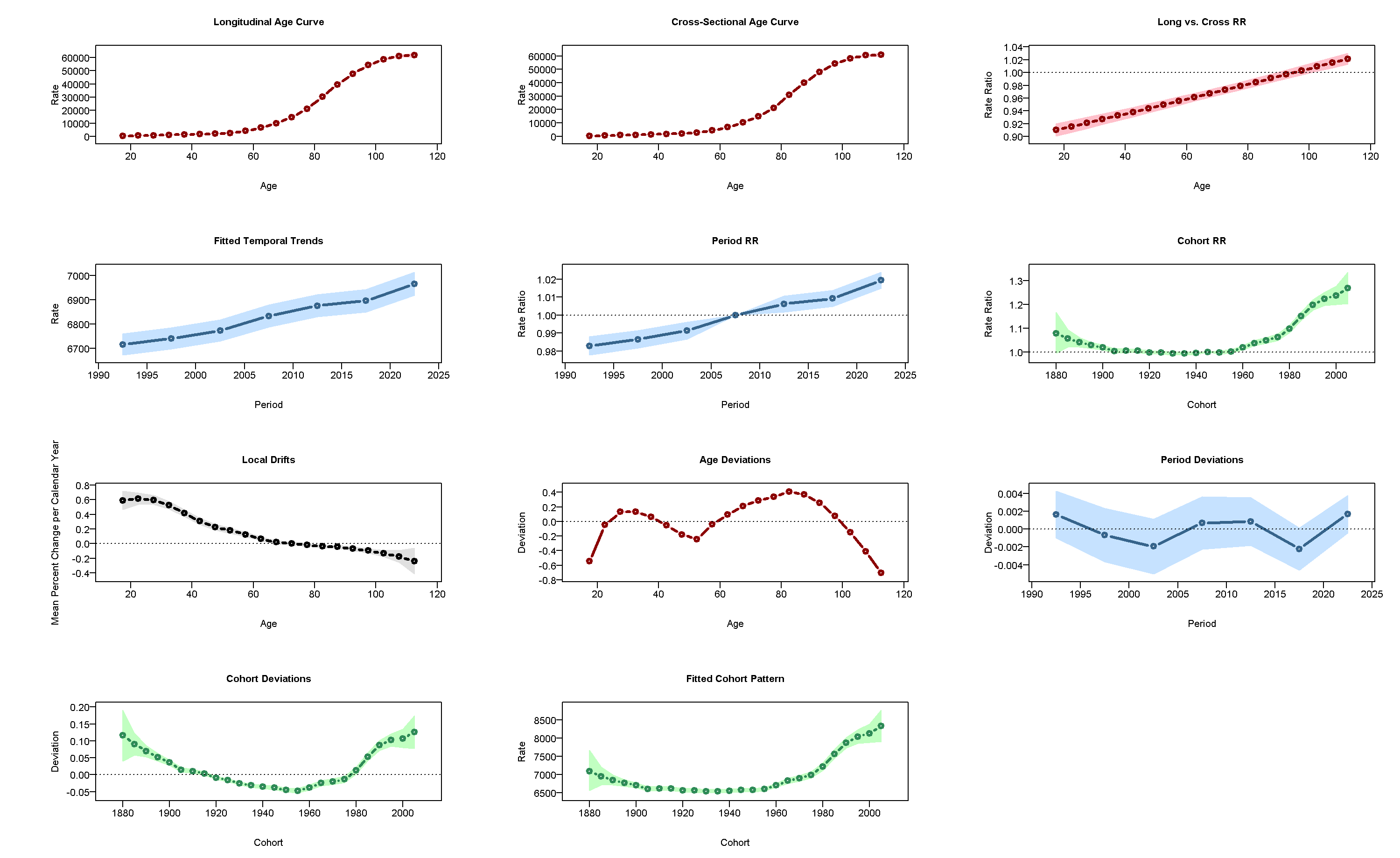

Supplement: Supplementary file 1 [file Datasheet1.zip › Supplementary Materials_Revised_2/Figure S1-S5/Figure S2.png]

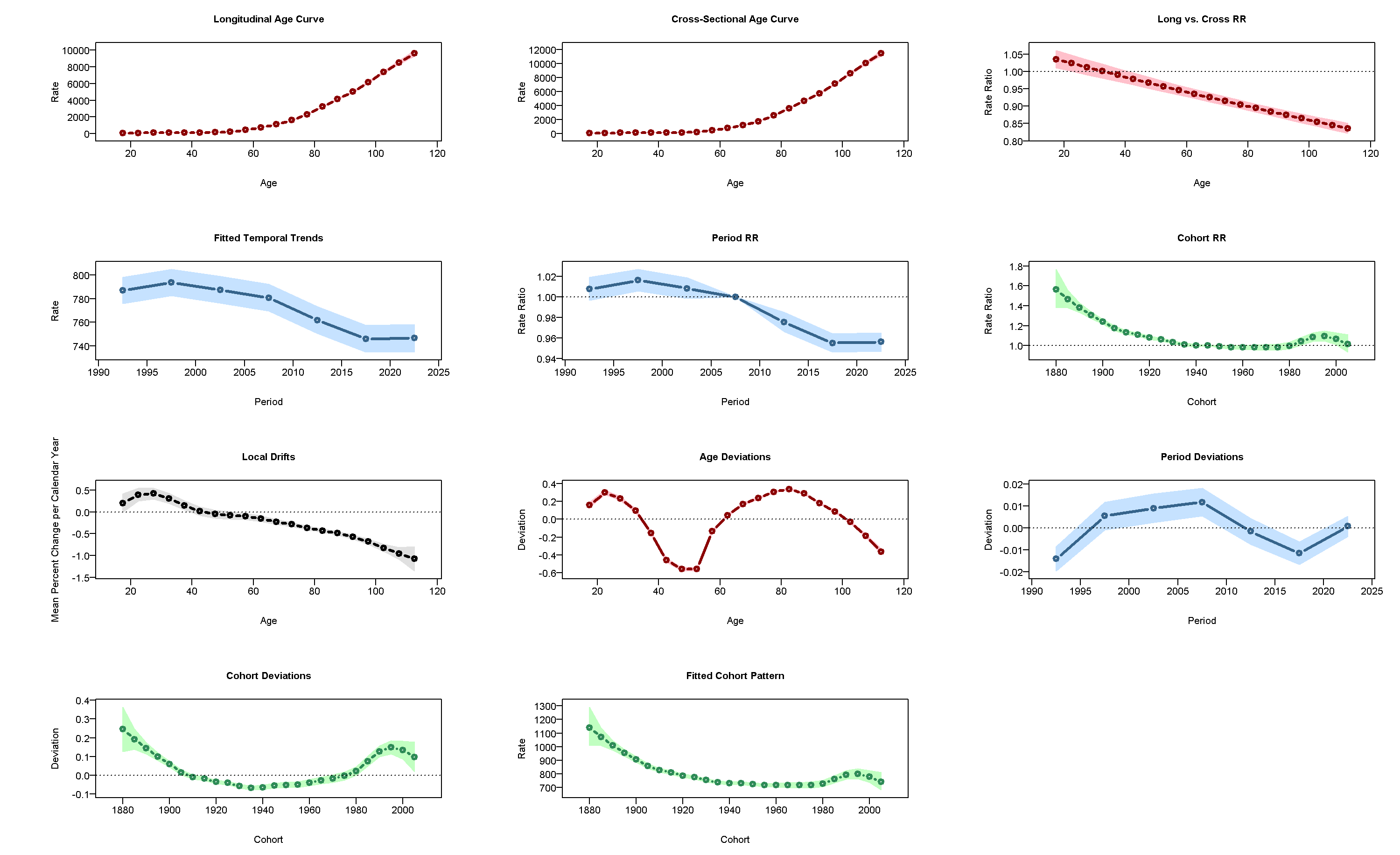

Supplement: Supplementary file 1 [file Datasheet1.zip › Supplementary Materials_Revised_2/Figure S1-S5/Figure S3.png]

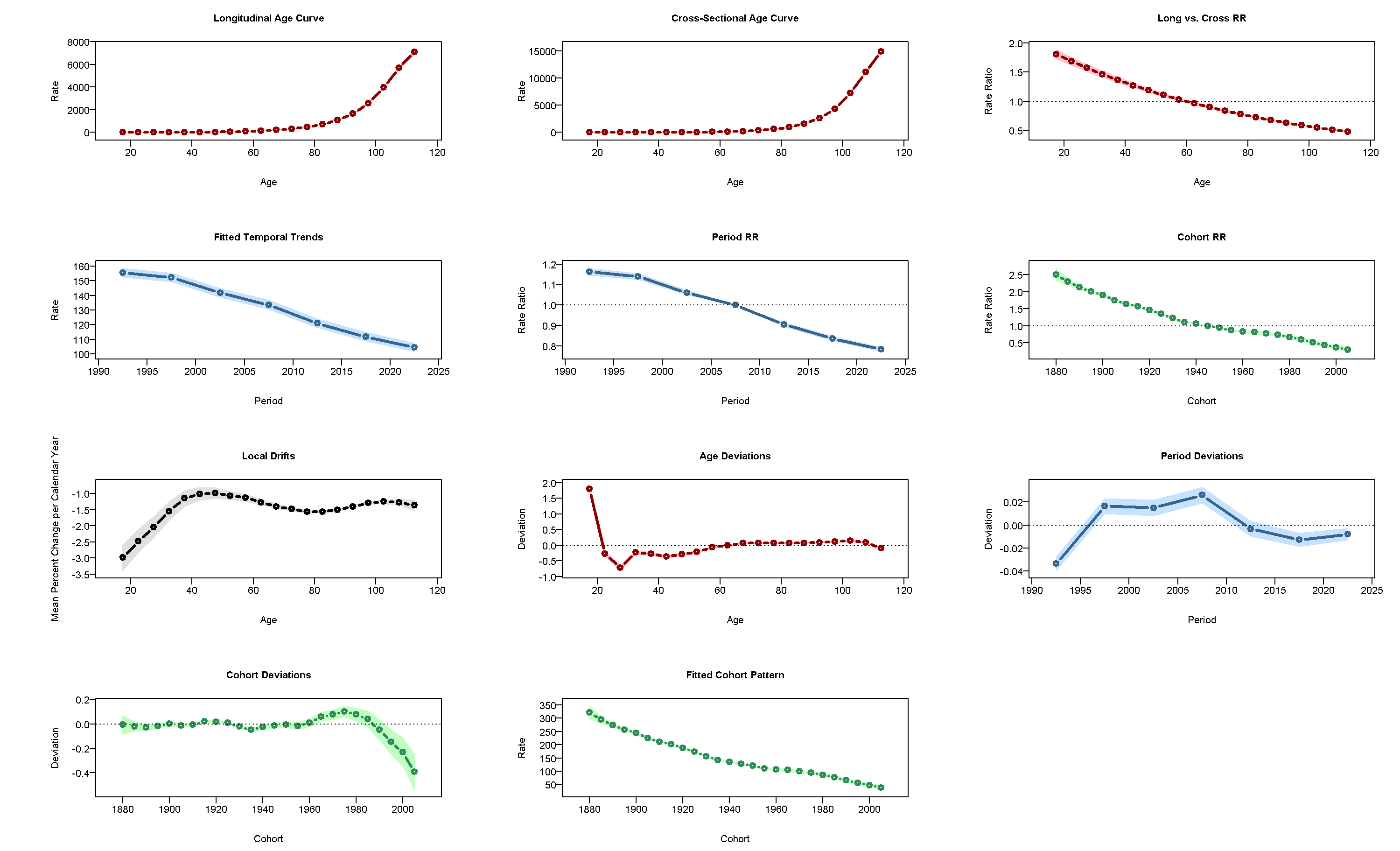

Supplement: Supplementary file 1 [file Datasheet1.zip › Supplementary Materials_Revised_2/Figure S1-S5/Figure S4.png]

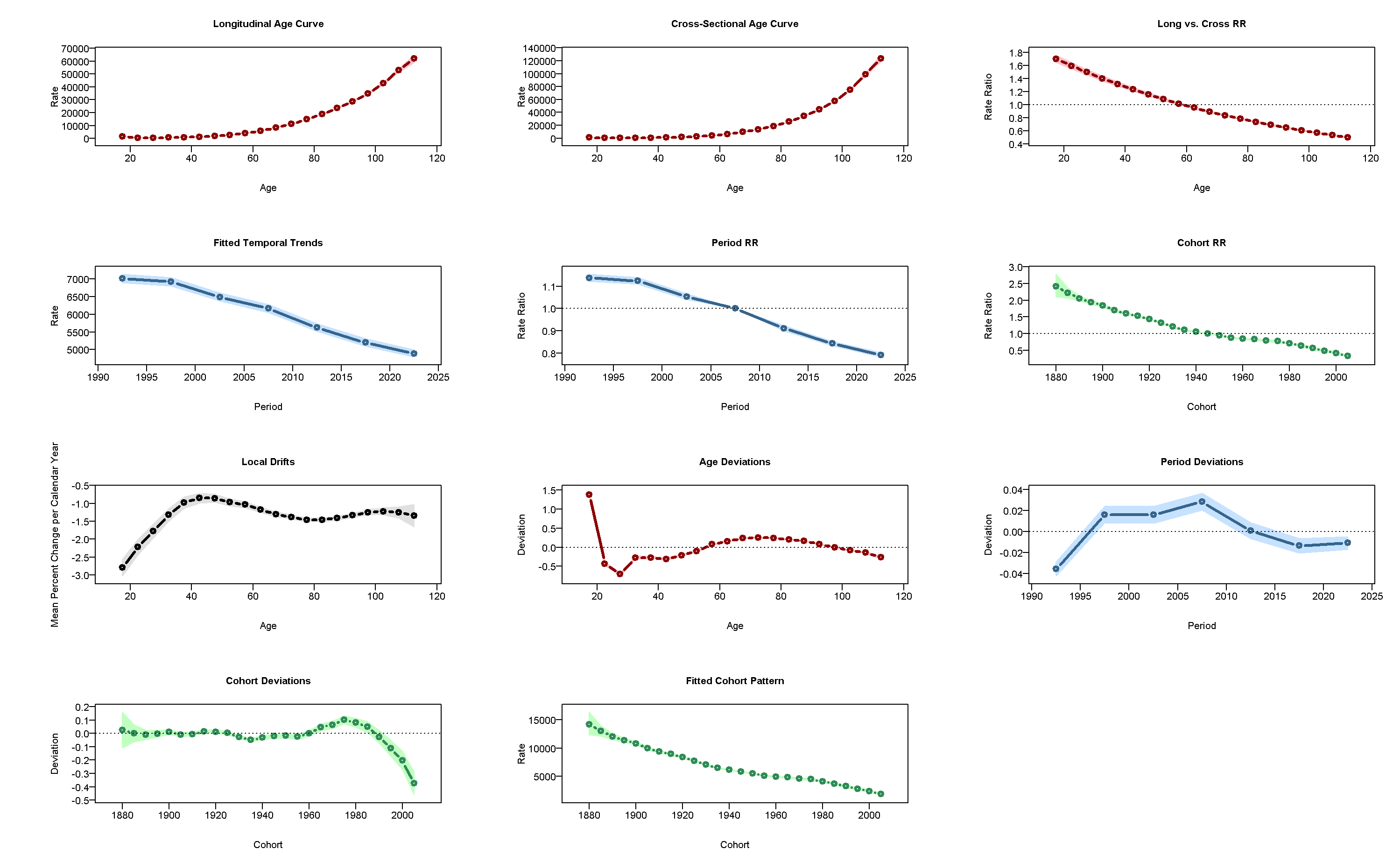

Supplement: Supplementary file 1 [file Datasheet1.zip › Supplementary Materials_Revised_2/Figure S1-S5/Figure S5.png]

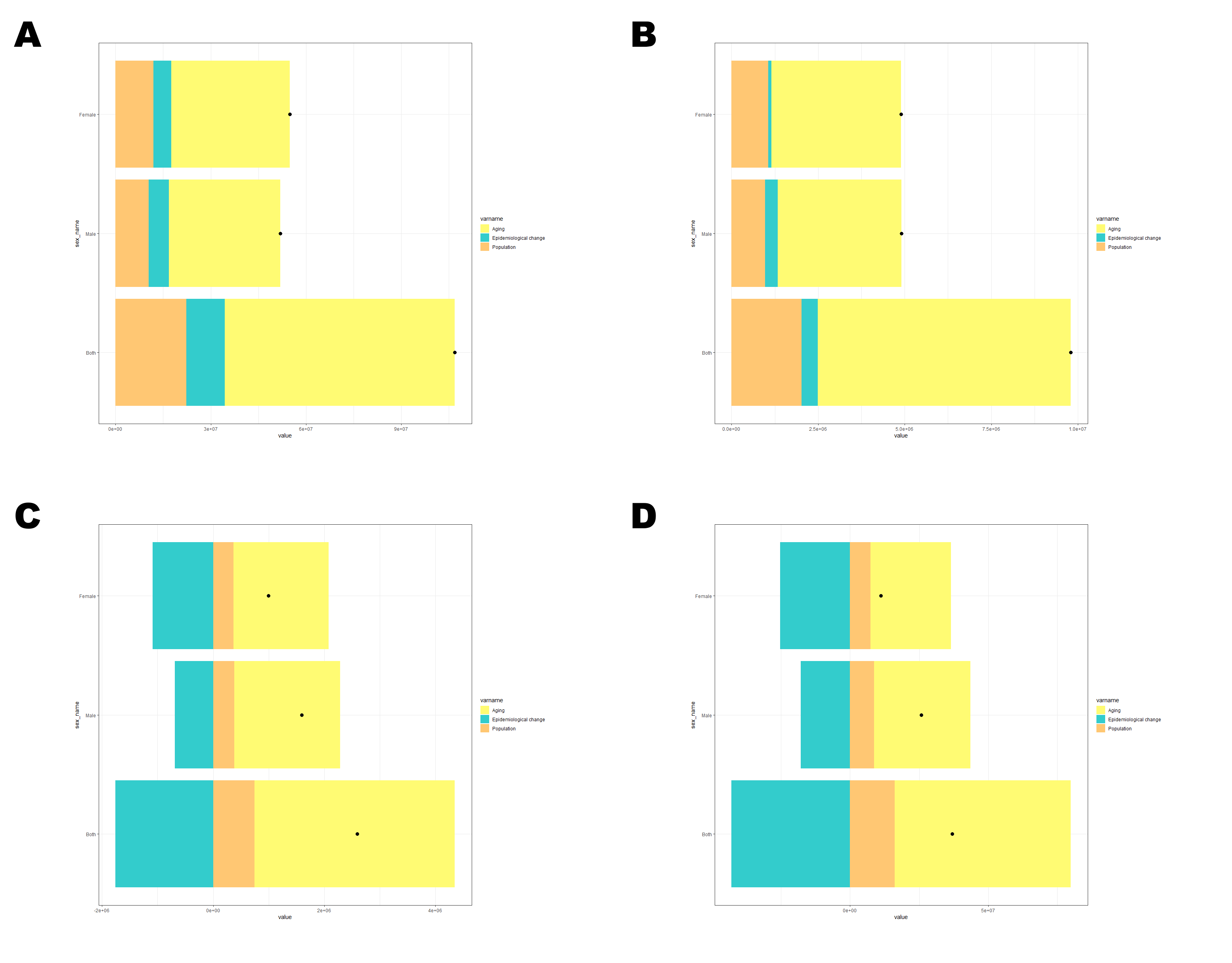

Supplement: Supplementary file 1 [file Datasheet1.zip › Supplementary Materials_Revised_2/Figure S6-S7/Figure S6 A-D.png]

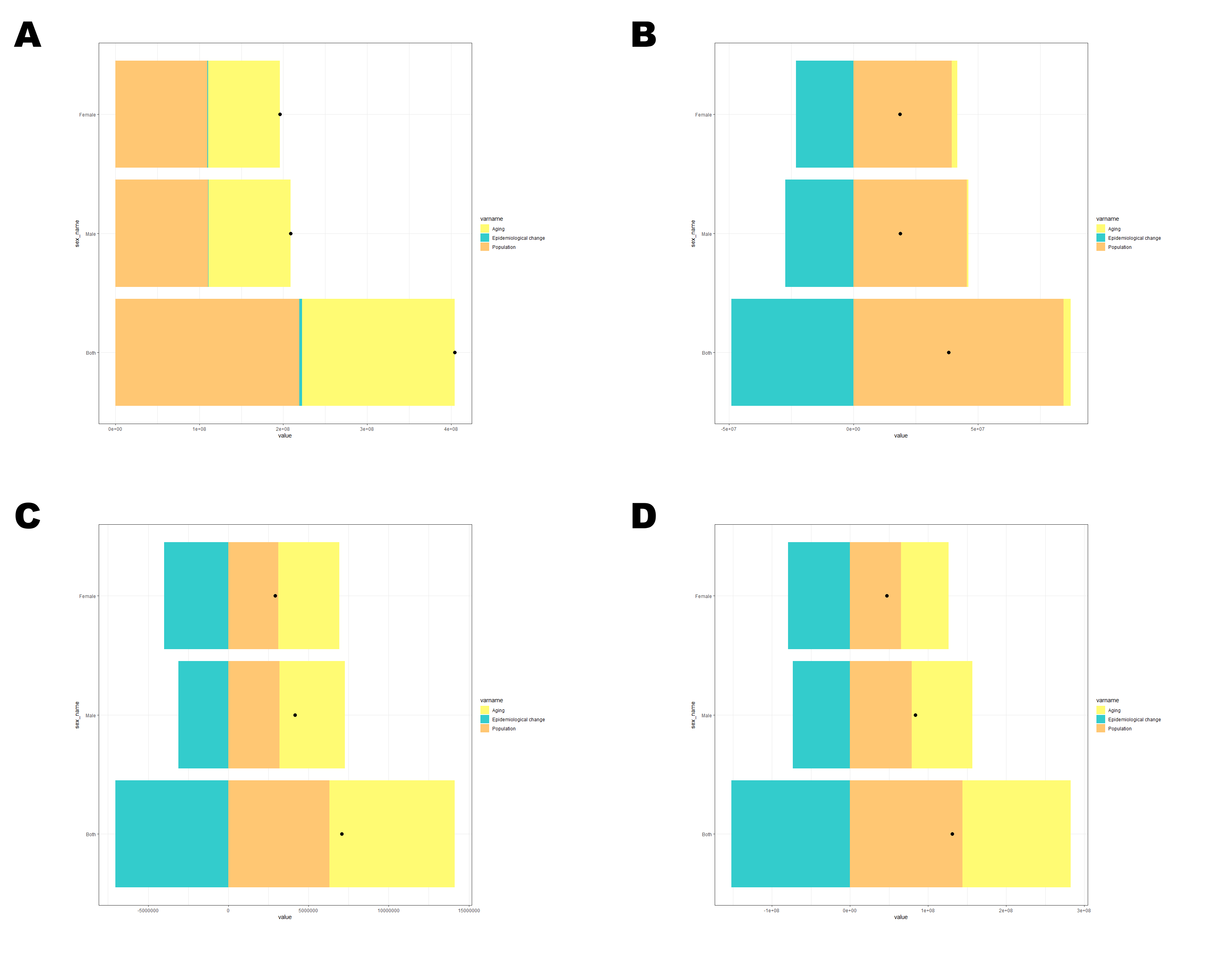

Supplement: Supplementary file 1 [file Datasheet1.zip › Supplementary Materials_Revised_2/Figure S6-S7/Figure S7 A-D.png]

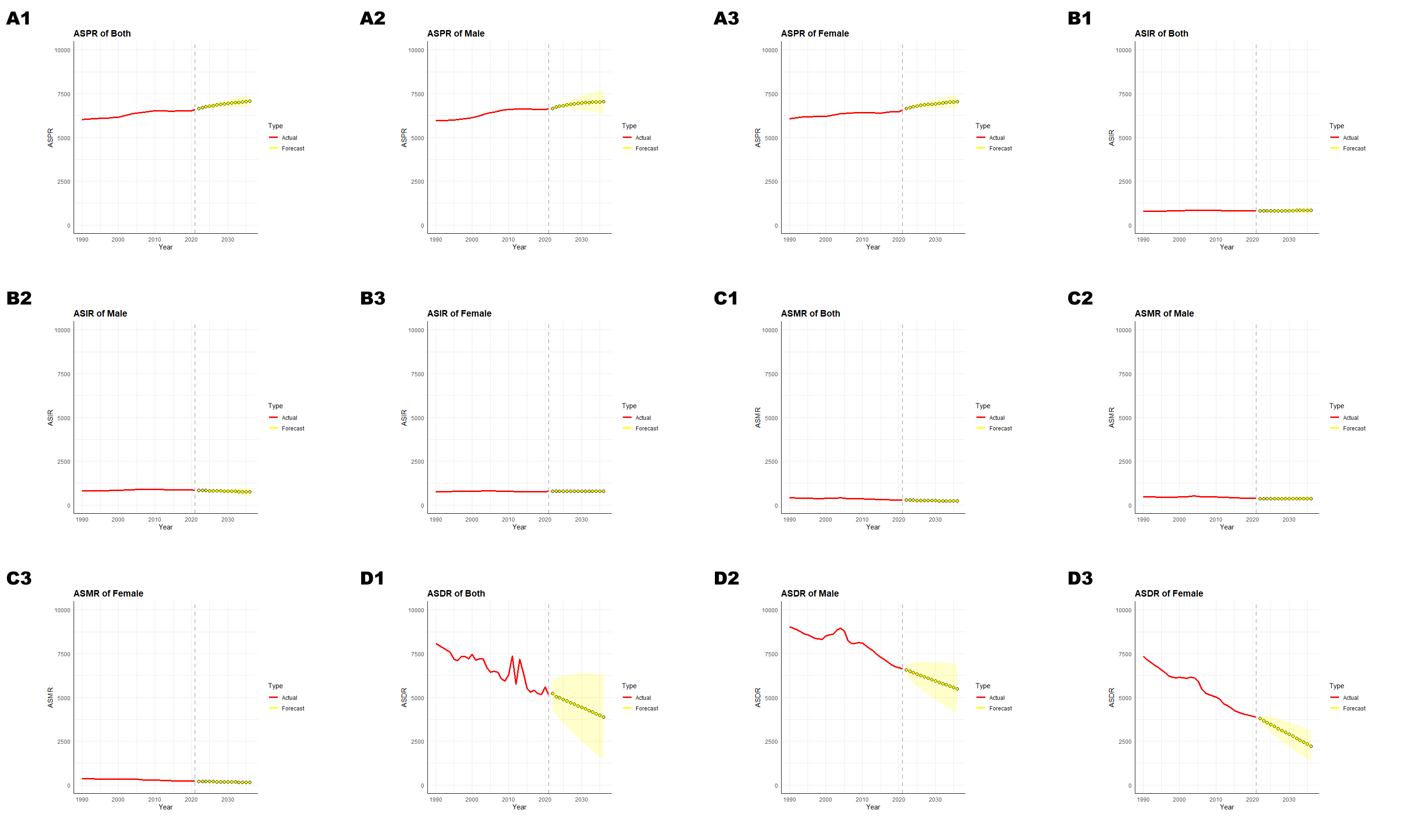

Supplement: Supplementary file 1 [file Datasheet1.zip › Supplementary Materials_Revised_2/Figure S8/Figure S8 A1-D1+A2-D2+A3-D3.png]

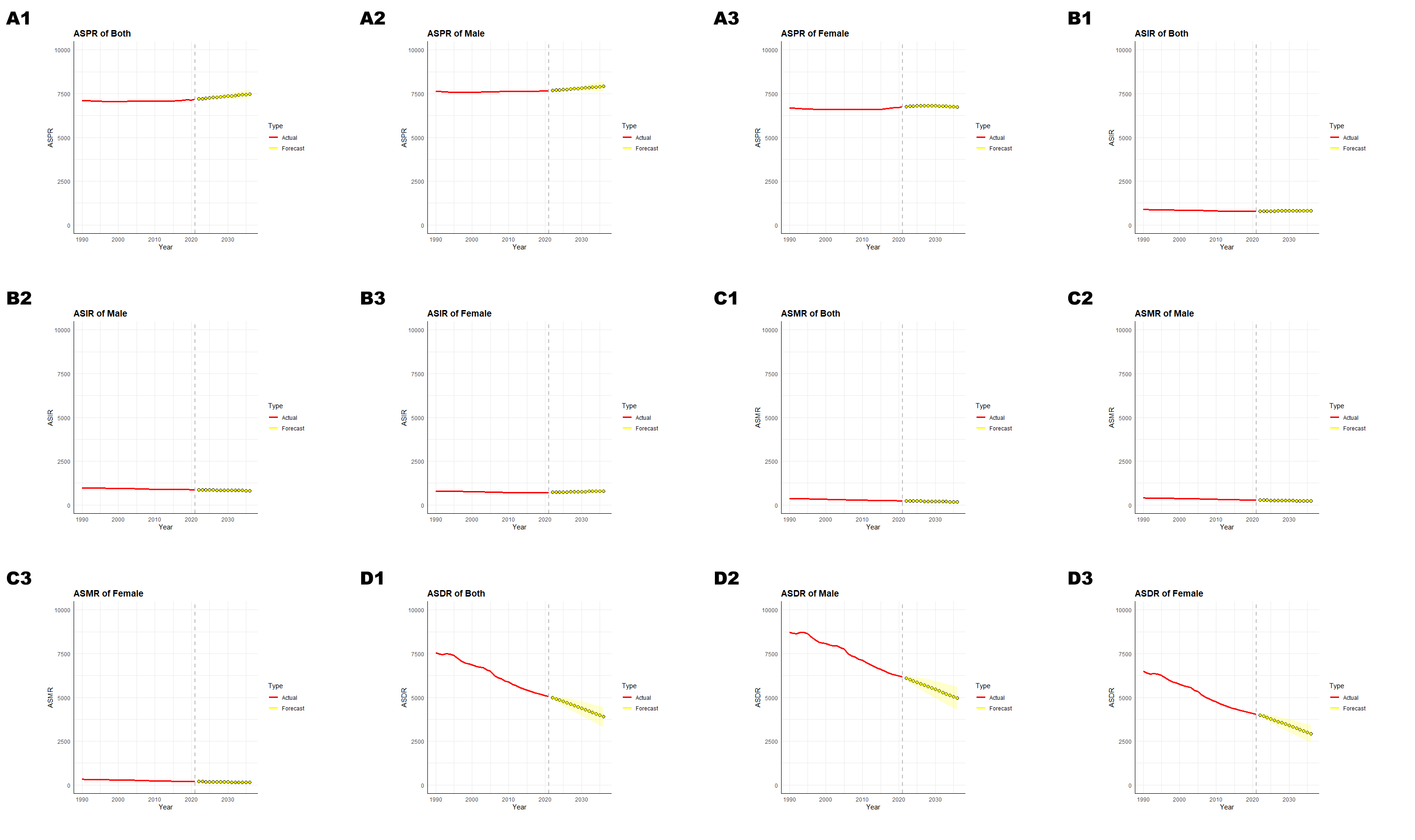

Supplement: Supplementary file 1 [file Datasheet1.zip › Supplementary Materials_Revised_2/Figure S9/Figure S9 A1-D1+A2-D2+A3-D3.png]
